# Rethinking Meat Alternatives in Eastern Europe: A Just Transition Lens on Policy, Perception, and Innovation in Romania

**DOI:** 10.1002/fsn3.71650

**Published:** 2026-04-24

**Authors:** Ruxandra Malina Petrescu‐Mag, Camelia Ginsca, Ioana Pistea, Kinga Olga Reti, Cristina Bianca Pocol, Radu Cristian Maricut, Dacinia Crina Petrescu

**Affiliations:** ^1^ Faculty of Environmental Science and Engineering Babes‐Bolyai University Cluj‐Napoca Romania; ^2^ Doctoral School “International Relations and Security Studies” Babes‐Bolyai University Cluj‐Napoca Romania; ^3^ Department of Economy and Rural Development, Faculty of Gembloux Agro‐Bio Tech University of Liège Gembloux Belgium; ^4^ Doctoral School of Environmental Science Cluj‐Napoca Romania; ^5^ Department of Animal Production and Food Safety University of Agricultural Sciences and Veterinary Medicine of Cluj‐Napoca Cluj‐Napoca Romania; ^6^ Faculty of Business Babes‐Bolyai University Cluj‐Napoca Romania

**Keywords:** alternative proteins, cultural acceptability, cultured meat, decision‐makers, food transition, insects, plant‐based

## Abstract

Alternative meat products, including plant‐based, insect‐based products, and cultured meat, are gaining momentum as sustainable food innovations. Yet, the perceptions of political and institutional actors, key players in shaping regulation, legitimacy, and public discourse, remain poorly understood. This study fills this gap by examining how Romanian policymakers and public institutional actors understand sustainable protein transition using the SPECT framework (Social, Policy, Environmental, Commercial, Technological dimensions). Using qualitative thematic analysis of semi‐structured interviews with 18 political and institutional actors, the research provides the first in‐depth account of how governance elites in an Eastern European context interpret the sustainability, risks, and legitimacy of meat alternatives. The originality of this research lies in reframing political and institutional actors as active co‐authors, rather than passive implementers, of sustainable food transition. The findings reveal a cautious but structured hierarchy of acceptance: plant‐based products are perceived as “palatable bridges” to sustainability, whereas insect‐based and cultured meat products provoke reactions ranging from curiosity to strong symbolic rejection. Concerns extend beyond health or naturalness to include food sovereignty, fairness for domestic producers, and the preservation of culinary identity. Findings highlight the concept of trust as the “currency of transition,” showing that acceptance depends on credible institutions, transparent certification, and communication strategies grounded in local food traditions rather than globalist or “eco‐elitist” narratives. Within the SPECT framework, social and policy dimensions proved more influential than technological or commercial ones, highlighting the primacy of cultural identity and institutional trust in shaping actors' perceptions. By situating Romania's case within the broader debate on just and inclusive food transition, the research provides new empirical evidence on how national identity, smallholder farming structures, and policy skepticism influence the political feasibility of adopting meat alternatives. The study concludes that aligning innovation with national food priorities through trust‐based, inclusive policy design is essential for enabling a culturally embedded protein transition. Although the findings offer insights relevant to broader debates on sustainable protein transitions, their transferability to other national contexts is limited by Romania's specific cultural, institutional, and agri‐food structures.

## Graphical abstract

The graphical abstract illustrates key elements in the perceptions of Romanian policymakers and public institutional actors (derived from qualitative research) regarding sustainable protein transition, structured according to the SPECT framework (Social, Policy, Environmental, Commercial, Technological dimensions).

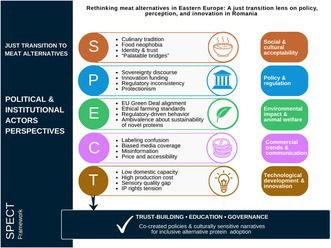

## 1 Introduction

### 1.1 The Sustainability Crisis in Global Food Systems

Food systems worldwide face an urgent sustainability challenge as the global population approaches 9.7 billion by 2050 (Malila et al. 2024). Meeting rising food demand with conventional animal agriculture is increasingly untenable because of its heavy environmental footprint. Livestock farming contributes an estimated 14.5% of anthropogenic greenhouse gas emissions (Bateki et al. 2023; Thavamani et al. 2020) and drives deforestation, biodiversity loss, and water scarcity through the cultivation of feed and the expansion of pastures (Govoni et al. 2023). Global livestock production was estimated to consume approximately 2422 km^3^ of water annually, accounting for around one‐third of the total agricultural water use, and about 98% of this consumption was linked to the water required for producing animal feed (Mekonnen and Hoekstra 2012). Intensive animal production also raises concerns about zoonotic disease risks and antibiotic resistance (Stevenson 2023; UNEP 2020). In response, there is growing interest in sustainable food systems that can provide sufficient nutrition while curbing climate change and resource depletion.

Nonetheless, we acknowledge that animal agriculture also plays a vital role in global food systems by providing high‐quality protein and essential micronutrients, particularly in regions where plant‐based alternatives are not readily accessible (Mottet et al. 2017). Moreover, livestock contributes to the livelihoods of over 1.3 billion people worldwide and can support circular agriculture through the recycling of crop residues and manure (FAO 2018). Recognizing both its benefits and drawbacks is crucial for shaping balanced and sustainable food policy.

Although these global dynamics are well documented, the governance dimensions of protein transitions remain comparatively underexplored. The shift toward sustainable proteins is not merely a technological innovation, but a governance challenge that hinges on how political and institutional actors interpret and legitimize it. Their perceptions shape regulation, public funding, and communication strategies, ultimately determining the speed and social acceptance of food‐system transformation (Leeuwis et al. 2021). However, these actors' views remain marginal in the literature compared with the extensive scholarship on consumers, markets, and technological performance. Addressing this gap is a central focus of the present study.

### 1.2 The Promise and Pitfalls of Meat Alternatives

A key pillar of this transformation is the development of meat alternatives, notably plant‐based meat analogues, cultured meat, and insect‐based foods, which are promoted as more environmentally friendly and ethical protein options (UNEP 2023). These innovations hold the promise of reducing land use, water consumption, and greenhouse emissions relative to conventional meat while also advancing animal welfare by decoupling protein production from livestock slaughter (Kumar et al. 2023; Thornton et al. 2023). Alternative meat products encompass a spectrum of technologies and ingredients. Plant‐based meat alternatives (made from soy, pea, wheat gluten, etc.) are the most mature segment, already achieving notable market acceptance and rapid growth (Malila et al. 2024). From a health perspective, they often provide nutritional advantages, support weight management and muscle growth, and can be beneficial for individuals with specific health needs (Bryant 2022). However, they are still often considered inferior to animal proteins because of lower digestibility, suboptimal essential amino acid profiles, and less favorable techno‐functional and sensory properties (Munialo and Vriesekoop 2023).

Considering the environmental impact, soy‐based meat alternatives have a 4–20 times lower environmental impact than beef (Herrmann et al. 2024). Yet, some studies (Aimutis and Shirwaiker 2024; Herrmann et al. 2024) highlight that the processing of plant proteins, such as extraction and concentration, can contribute to their environmental footprint, although this is generally still less than that of animal‐based meats. In this study, the term “conventional meat” is preferred over “traditional meat,” which is often used in scientific literature to describe meat from animals raised and processed through standard farming methods. This choice helps avoid confusion with “traditional products,” a specific food category in Romania referring to officially labeled items made according to traditional recipes, ingredients, or production techniques.

Insect farming requires far less land, water, and feed, and emits substantially lower greenhouse gases, making it a resource‐efficient alternative that supports circular economy models by recycling organic waste into high‐quality protein and beneficial fats (Li et al. 2023; Lisboa et al. 2024). However, it is worth noting that a study by Biteau et al. (2025), which highlights critical flaws in insect farming research, noting that many studies depend on outdated sources to evaluate environmental impact. It also questions the common assumption that insect farms will primarily use food waste as feed and challenges optimistic price estimates that ignore real‐world commercial constraints. These aspects raise concerns about the reliability of current sustainability claims of insect farming. Meanwhile, cultured meat, cultivated from animal cells in bioreactors, is an emerging technology that aims to replicate the taste and texture of real meat without requiring the raising or slaughtering of animals. Sinke et al. (2023) consider that cultured meat has the potential to have a lower environmental impact than conventional meat production by 2030, with a carbon footprint substantially lower than that of beef and comparable to that of chicken. Still, controversies are present. Some research reports that producing cultured meat results in a considerable carbon footprint, primarily because of the energy demands of maintaining controlled environments for cell cultivation (Smetana et al. 2015). Additionally, questions remain about the long‐term sustainability of large‐scale production, as well as challenges related to food safety and consumer acceptance (Stephens et al. 2018).

Although this literature offers critical insights into technological potential and consumer behavior, it rarely considers how policymakers and institutional actors (who design, regulate, and fund these developments) frame their opportunities and risks. Their perspectives shape not only national policy trajectories but also public narratives around food sovereignty, fairness, and cultural continuity. Understanding these perceptions is therefore vital for explaining how sustainable protein transitions unfold in practice. Taken together, these debates highlight that the future of alternative proteins is shaped not only by innovation trajectories, but also by how they are interpreted, regulated, and legitimized by political and institutional actors.

### 1.3 Social Acceptance and the Need for a Just Transition

This study, therefore, shifts the analytical focus from products and consumers to the institutional actors who actively construct the social and political legitimacy of emerging protein technologies. This shift is particularly relevant given that, despite their potential benefits, meat alternatives currently account for only a small fraction of diets, and their expansion faces significant social, technical, and regulatory challenges (Malila et al. 2024; UNEP 2023). One significant barrier is social and cultural acceptability. Food choices are deeply rooted in culture and tradition, and many consumers view novel meat substitutes with skepticism or even disgust. In Europe, including Romania, entomophagy (eating insects) is associated with a pronounced “yuck factor” (Andreani et al. 2024). A Romanian study (Petrescu‐Mag et al. 2022) found that “disgust,” closely related to the fear of contamination (Jensen and Lieberoth 2019), was a frequent reaction to the idea of eating insects, reflecting low cultural tolerance for such unfamiliar foods.

Similarly, cultured meat and fermentation‐derived proteins are often perceived as “unnatural” or highly artificial, which can provoke consumer hesitancy or ethical concerns (Akinmeye et al. 2024). Although greater knowledge and transparency can improve openness (Petrescu‐Mag et al. 2022), recent evidence from Romania suggests that sustainability labels alone have limited capacity to overcome such deeply embedded cultural perceptions (Petrescu et al. 2025).

Romania is a traditionally meat‐centric society, and many Romanians adhere to culinary norms that leave little room for unconventional ingredients (Petrescu‐Mag, Pistea, et al. 2025). These reactions are not merely informational deficits but reflect broader identity, trust, and moral boundaries surrounding what counts as “real food.” However, studies reported willingness among Romanian consumers to reduce meat consumption and waste, suggesting potential to increase diet sustainability in this direction (Petrescu et al. 2017). Ensuring that meat alternatives respect local gastronomic heritage (e.g., adapting products to familiar flavors or dishes) and moral values (e.g., religious ones; Petrescu‐Mag et al. 2020) or focus on food waste reduction (Petrescu‐Mag et al. 2019) may also facilitate cultural integration (Koponen et al. 2023).

However, social acceptance alone is insufficient. The transition to sustainable proteins also depends on the institutional legitimacy granted by policymakers and public authorities who set regulatory priorities and shape communication agendas (Bulah et al. 2023). These actors determine whether alternative proteins are integrated into national food strategies or pushed as niche innovations. Their framing of sustainability, whether in terms of opportunity, threat, or cultural loss, becomes a central determinant of transition outcomes. This study explicitly addresses this underexplored dimension by investigating how Romanian political and institutional actors perceive these emerging food technologies.

Recognizing and addressing these cultural perceptions is essential not only for consumer acceptance but also for a fair and inclusive transformation of the food system. This is where the concept of a “just transition” becomes particularly relevant, emphasizing that sustainability efforts must align with societal values and avoid excluding or alienating communities in the process. Thus, the concept of “just transition” in food systems emphasizes that shifts toward sustainability must be socially inclusive and culturally sensitive, thereby avoiding alienating consumers or dismissing their values. Originating in the 1980s labor movements, “just transition” is defined by UNDP (2022) in accordance with ILO principles as “greening the economy in a way that is as fair and inclusive as possible to everyone concerned, creating decent work opportunities and leaving no one behind.”

In the context of sustainable food systems, a just transition emphasizes fairness in who benefits from and who bears the costs of food system changes, particularly for vulnerable populations (UNEP 2023). As highlighted in recent studies, a sustainable food system should not only meet environmental goals but also address economic viability and social equity (de Bruin et al. 2024; Keefe and Lee 2025). This includes supporting cultural food preferences, regional livelihoods, and equitable access to innovation. For instance, consumer attitudes toward meat alternatives are shaped by trust, tradition, and familiarity, which must be respected in any meaningful transition (Akinmeye et al. 2024; Petrescu‐Mag, Pistea, et al. 2025). Therefore, achieving a just transition toward a sustainable food system involves aligning technological advancements with local contexts, values, and voices.

One key dimension of a just transition is the policy and regulatory framework necessary to guide it. Policymakers face a dual challenge: to incentivize sustainable protein innovation and consumption, while avoiding undue harm to conventional meat producers and rural livelihoods. In Romania, as in many countries, animal agriculture is a significant sector that supports farmers and the food industry. A sudden shift away from meat could threaten economic and social stability if not managed carefully. Thus, a just transition approach calls for policies that support farmers in diversification and retraining, providing financial incentives to adopt alternative protein production or more sustainable practices, rather than simply penalizing traditional producers (UNEP 2023). Tools such as subsidies, tax breaks, or R&D grants could encourage startups and food companies to develop affordable meat alternatives (Kristiansen et al. 2021). Measures such as environmental taxes on high‐emission meat or redirecting farm subsidies toward protein‐rich crops might gradually rebalance the market. However, such measures must be calibrated to ensure equity across social, economic, and environmental dimensions. The role of institutions and decision‐makers is therefore essential in designing win‐win policies that drive innovation (Hundscheid et al. 2022) without positioning sustainable proteins against conventional agriculture in a zero‐sum game.

Developing domestic supply chains for alternative meat products can significantly enhance food sovereignty by increasing local control over food production and reducing reliance on global, industrial supply chains. Short supply chains and local food systems enable communities to make informed decisions about how food is produced and distributed, fostering resilience, transparency, and self‐sufficiency while supporting sustainable practices (Byaruhanga and Isgren 2023; Kapała 2023). This is particularly relevant for Romania, where a high proportion of smallholder farmers [Romania has the EU's largest farming population, with 90% of farms under 5 ha (European Commission 2025)] and a national policy focus on food self‐sufficiency align closely with these principles. These characteristics make Romania a strategically relevant case, as it combines strong agricultural traditions and a cultural attachment to meat that intersect with debates on sovereignty, innovation, and EU integration. Examining this context can shed light on how sustainability narratives are shaped by political identity, institutional capacity, and trust. Although governance research on alternative proteins has largely focused on Western European and North American contexts (e.g., Bulah et al. 2023; Hundscheid et al. 2022), it has rarely captured the post‐communist institutional legacies, high prevalence of smallholders, and persistent policy skepticism that characterize Central and Eastern European (CEE) countries. The Romanian case, therefore, offers analytically transferable insights into how institutional trust, cultural identity, and state capacity jointly mediate food transitions across the wider CEE region, rather than representing a purely idiosyncratic national context.

### 1.4 SPECT Framework, Research Questions, and Objective

As already presented, to guide Romania toward a fair and effective protein transition, it is essential to understand not only the environmental benefits of meat alternatives but also the broader social, cultural, economic, and institutional factors that shape their adoption. Thus, achieving a just transition toward sustainable meat alternatives requires an integrated approach that addresses the full spectrum of societal, institutional, and technological factors, precisely what the SPECT framework captures.

*Social and cultural acceptability (S)* is foundational, as food is deeply tied to cultural identity, tradition, and trust; overlooking this can provoke public resistance and erode support for sustainability initiatives (Akinmeye et al. 2024; Petrescu‐Mag, Pistea, et al. 2025). *Policy and regulation* dimension *(P)* provides structural levers to guide the transition through fiscal tools, food safety rules, and strategic governance. Still, they must be calibrated to avoid penalizing conventional farmers and exacerbating rural inequalities. As Hundscheid et al. (2022) argue, institutions must shift beyond technological optimism to reshape norms, incentives, and public discourse that currently uphold meat‐centric systems. Without this systemic change, the sector of meat alternatives will remain peripheral. *Environmental and animal welfare concerns (E)* are central to the moral and scientific case for alternatives, particularly in relation to climate targets and growing ethical concerns about industrial livestock (Kumar et al. 2023). *Commercial and communication (C)*, such as labeling, branding, and public narratives, influence consumer behavior, trust, and perceived legitimacy, reinforcing or challenging existing dietary patterns (Kristiansen et al. 2021; Malila et al. 2024). *Technological innovation (T)* plays a crucial role in reducing production costs, enhancing product realism, and establishing resilient supply chains. However, as Hundscheid et al. (2022) outline, technological solutions must be complemented by institutional reform to prevent reinforcing existing power imbalances or techno‐centric biases.

**FIGURE 1:**
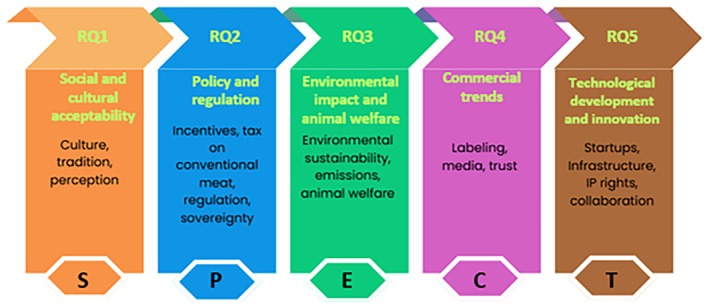
SPECT framework for a just transition to alternative meat products.

The SPECT framework is employed here not as a novel theoretical model but as an integrative analytical heuristic that allows a systematic exploration of how these five dimensions interact to shape institutional perceptions. It operationalizes the principles of the just transition by bridging social, political, and technological domains, offering a pragmatic lens to capture the interdependencies that condition food‐system change. The SPECT framework (Figure 1) thus provides a comprehensive lens for examining Romania's transition to meat alternatives, ensuring that it is not only environmentally effective but also socially inclusive, economically viable, and culturally appropriate. Accordingly, the study's objective is to examine how Romanian political and institutional actors perceive the drivers, constraints, and policy implications of transitioning to sustainable proteins, and to identify how these perceptions shape pathways toward a culturally grounded and socially inclusive food transition. Using the SPECT framework, this study addresses the following research questions (RQ).

*What are the social and cultural factors influencing the acceptability of alternative meat products (cultured meat, insect‐based products, and plant‐based products) in Romania?*

*How can fiscal and regulatory policies encourage the adoption of meat alternatives without disadvantaging conventional meat producers?*

*What impact do international and EU food safety regulations have on the development of alternative meat products in Romania, and how do they affect national food sovereignty?*

*What measures can be taken to develop a sustainable food system in Romania that balances environmental protection and animal welfare?*

*How can labeling practices for alternative meat products in Romania be improved to ensure transparency, prevent consumer confusion, and support informed food choices?*

*What role does media coverage, across television, social media, blogs, and political discourse, play in shaping Romanian consumers' perceptions, attitudes, and trust toward alternative meat products?*

*What technological and infrastructural challenges hinder the growth of the alternative meat sector in Romania, and how can they be addressed?*

*What role do intellectual property rights and cross‐sector collaboration play in fostering or limiting innovation in Romania's alternative meat industry?*

By addressing these questions, the study positions political and institutional actors as active co‐authors, rather than passive implementers of sustainable food transitions, whose perceptions shape the legitimacy, governance, and operational pathways of Romania's protein transition. In doing so, it moves beyond instrument‐focused governance studies by empirically demonstrating how legitimacy is socially constructed through institutional trust, national identity, and policy narratives. More broadly, it contributes to food transition governance theory by showing how state capacity, cultural identity, and institutional skepticism condition the political feasibility of sustainability transitions in post‐communist and smallholder‐dominated contexts.

## 2 Methodology

### 2.1 Participant Selection

This study focused on Romanian decision‐makers and politically engaged individuals (hereinafter referred to as “political and institutional actors”), a category that includes both those employed in public organizations and individuals who, although not directly influencing, shaping, or implementing public policies, are actively involved in the structures of political parties. A qualitative research design was employed, using semi‐structured interviews (the interview questions are available in Table S1, Appendix S1) to explore these actors' perceptions of meat alternatives and the transition toward a more sustainable food system.

In the initial recruitment phase, a purposeful sampling approach [as defined by Patton 2002] was used. Purposeful sampling, often used in qualitative research, identifies and selects individuals or groups who possess substantial knowledge or experience concerning the topic of interest (Palinkas et al. 2015). For this stage, we targeted Romanian members of the European Parliament and initiators of a legislative proposal about the ban on cultured meat. Additionally, relevant public authorities were approached, including, for example, the Ministry of Agriculture and Rural Development, the National Veterinary Sanitary and Food Safety Authority, functioning as the regulatory authority for veterinary and food safety under the Government of Romania, the National Commission for Novel Foods, and other governmental agencies with competencies in food safety, agricultural policy, health policy, or animal husbandry.

Invitations were sent by email and/or through various media platforms. Despite these extensive efforts, responses were limited. Only one Romanian European Parliament member replied directly, explaining that the topic fell outside their professional background. Consequently, a snowball sampling strategy (Noy 2008) was adopted to extend the participant pool. In this phase, participants were selected on the basis of their affiliation with a political party and active involvement in the party's activities (e.g., participation in meetings), or on their employment within public authorities connected to the food system. This method involved leveraging existing contacts active in political structures, who then recommended further potential interviewees from their professional or political networks. Snowball sampling is particularly appropriate for reaching specialized or hard‐to‐reach populations where official contact attempts have limited success (Atkinson and Flint 2001; Noy 2008). It was a challenging and time‐consuming process, given the political sensitivity of the topic of meat alternatives and the highly controversial nature of Romania's existing legislative proposal to ban cultured meat. Recruitment was therefore concluded because of a combination of access constraints, time limitations, and the evident reluctance of many political and institutional actors to engage with this topic, as further outreach efforts yielded diminishing responses and proved unfeasible within the project timeframe. Nevertheless, we ultimately secured 18 interviews, which provided diverse insights from these actors. Following Saunders et al. (2018), saturation was assessed pragmatically and conceptually rather than numerically. Regarding data saturation, the study did not aim to reach saturation within individual political parties, as the analytical focus was not on intra‐party variation but on cross‐cutting perceptions and discursive frames related to sustainable protein transitions among political and institutional actors. Empirical observation indicated that after approximately 15 interviews, no substantially new themes, policy frames, or explanatory patterns emerged with respect to the study's core RQs. The final three interviews served to confirm and refine existing categories rather than generate new ones. In this sense, thematic saturation at the levels of perception and narrative was considered to have been achieved. At the same time, authors acknowledge that saturation within each political party was neither achievable nor intended, given the exploratory nature of the study, the limited access to political elites, and the uneven distribution of respondents across parties, a common challenge in qualitative research involving political actors (Vis and Stolwijk 2021).

All interviews were anonymized to protect participants' identities and in full compliance with data protection regulations, including the General Data Protection Regulation (GDPR). The study received ethical approval from Babes‐Bolyai University (Approval No. 7151, dated May 24, 2024). Participants were informed about the interview process, including the purpose of the research, and provided informed consent. They were also made aware of their rights, including confidentiality, privacy protection, and the option to withdraw from the interview at any point without needing to offer a reason. To ensure confidentiality, all interviews were anonymized, and any personally identifiable information was excluded from the final dataset. In the manuscript, participants were assigned neutral identifiers (“P” from “participant”) to safeguard their identities further.

It is essential to note that the opinions expressed by participants affiliated with public institutions or political parties are strictly personal and do not represent the official positions of the institutions or parties with which they are associated. Demographic characteristics of the participants are presented in Table 1.

**TABLE 1:** Demographics of participants.

Variable	Value
No. of participants	18
Average age	36.16 years old
Gender	80% men
Experience in public institutions/as party member (avg.)	7.5 years
Political party*	No. of participants
Social Democratic Party (Partidul Social Democrat, PSD^a^)	6
National Liberal Party (Partidul Național Liberal, PNL^b^)	1
Save Romania Union (Uniunea Salvați România, USR^c^)	3
Renewing Romania's European Project (Reînnoim Proiectul European al României, REPER^d^)	1
Democratic Alliance of Hungarians in Romania (Uniunea Democrată Maghiară din România, UDMR^e^)	3
Romanian National Conservative Party (Partidul Național Conservator Român, PNCR^f^)	1
Alliance for the Union of Romanians (Alianța pentru Unirea Românilor, AUR^g^)	1
Health, Education, Nature, Sustainability Party (Sănătate, Educație, Natură, Sustenabilitate, SENS^h^)	1
Independent (no party affiliation)	1

* Ideological position (Matache 2018): ^a^Center‐left party, which adheres to the Party of European Socialists, having a social‐democratic‐conservative ideology, pro‐EU; ^b^Center‐right party that adheres to the European People's Party (Christian‐democratic), with a liberal‐conservative ideology, pro‐EU; ^c,d^Center to center‐right, reformist, progressive party; ^e^Centrist regionalist party, affiliated with the European People's Party, with a liberal‐conservative ideology; ^f,g^National conservatism, Christian right, right‐wing populism; ^h^Center to center‐left, green‐progressive, pro‐sustainability, pro‐EU.

The demographic data above reflect a wide range of political affiliations among participants. Political ideology was not the study's core focus; however, party affiliation data were collected to enhance the contextual understanding of participants' responses. According to the latest INSCOP poll (Summer 2025), AUR leads in voting intentions with 40.5%, followed by PNL (17.3%), PSD (13%), and USR (13.1%). UDMR registers 5.2%, slightly up from 4.5% in May 2025, whereas SENS shows a modest decline to 2.4% from 3.3% (EuronewsRO 2025). These shifts mark significant changes from the December 2024 parliamentary elections.

### 2.2 Analytical Approach

The analytical process was based on thematic analysis (Braun and Clarke 2006), which enables the identification, analysis, and reporting of themes within qualitative data. Thematic analysis is particularly well‐suited for complex social issues, such as the sustainable food system, as it helps researchers to organize and interpret a rich set of perspectives systematically.

Within the thematic analysis framework, both deductive and inductive coding strategies were considered. Deductive coding involves applying a set of pre‐existing theoretical or conceptual codes to the data (Fereday and Muir‐Cochrane 2006). In contrast, inductive coding involves developing codes directly from the data without a predetermined coding frame, allowing patterns to emerge organically (Thomas 2006). In this study, a primarily deductive approach was chosen, informed by research questions derived from the interview guide, while remaining open to inductively identifying emergent subthemes throughout the analysis (see Table S1, Appendix S1). Therefore, to integrate these two approaches, we employed a hybrid strategy (Fereday and Muir‐Cochrane 2006) in which the initial codebook was deductively developed from the research questions and the SPECT framework, and then iteratively refined through inductive coding as new patterns emerged during close reading of the transcripts. This allowed theoretical alignment while maintaining openness to context‐specific insights. Codes and subthemes were continuously compared, merged, or expanded during analysis to ensure conceptual coherence (Nowell et al. 2017).

### 2.3 Development of the Coding Framework

The deductive codes used in this analysis were chosen on the basis of a set of research questions, one corresponding to each of the key dimensions explored, including social and cultural acceptability, policy and regulation, environmental impacts, technological development, just transition, consumer attitudes, and equity of access. These dimensions were collectively framed in the SPECT, the conceptual framework proposed for this study to organize and interpret the complex interactions surrounding the adoption of meat alternatives in Romania, as perceived by institutional and political actors. The interview script for this study was developed within the framework of the ERA‐NET project ERANET‐JPI‐HDHL‐ComMEATted, which aimed to explore how decision‐makers in partner countries perceive and navigate the transition to alternative meat. For the present case, the research questions were formulated to comprehensively explore the transition to a sustainable food system on the basis of meat alternatives, reflecting policy, cultural, technological, and economic dimensions relevant to Romanian society. These questions guided the construction of the interview guide, ensuring that each thematic area was addressed. This approach supported consistency and comparability across interviews.

The coding process was carried out following the work of Corbin and Strauss (2014), who emphasize that deductive codes should be transparently linked to research objectives and theoretical assumptions. The inclusion of methodological explanation alongside relevant literature was intentional, as the literature served not as a separate review but as a conceptual anchor to refine and validate the coding frame (see Fereday and Muir‐Cochrane 2006). Integrating theoretical grounding directly into the methodological narrative ensured transparency and epistemological coherence, linking coding choices explicitly to established debates.

In addition to these research‐derived codes, insights from the scientific literature informed the refinement of the coding frame, especially for topics related to sustainability, technological innovation, and animal welfare. This literature‐grounded refinement ensured that the coding system was aligned with contemporary debates and challenges in the alternative meat sector.

To ensure reliability and analytical rigor, the coding was conducted by two researchers who independently coded an initial subset of transcripts (about 30%) and then met to compare interpretations, resolve discrepancies through discussion, and refine the codebook. The 30% subsample was purposively selected to maximize heterogeneity in political affiliation and institutional background, ensuring that the double‐coded material covered the full thematic and ideological range of the dataset. Agreement was achieved through iterative consensus discussions, following best practice in reflexive thematic analysis. This process functioned as an inter‐coder calibration exercise (O'Connor and Joffe 2020), enhancing transparency and reducing subjective bias. The remaining transcripts were then coded by the lead researcher, following the agreed‐upon framework. All modifications to the coding scheme (including merging, renaming, or splitting codes) were documented along with short memos explaining the rationale for each decision. This record served as an audit trail, allowing the analytical process to be transparently traced from raw data to final themes (Nowell et al. 2017).

The development of deductive codes was inspired by a broad body of literature that explores multiple dimensions of sustainability, technological innovation, or animal welfare. To avoid analytical circularity, the literature was used exclusively to inform the initial deductive sensitizing concepts and not to validate or interpret empirical findings. All themes reported in the Results emerged from systematic coding of interview data and were assessed through inter‐coder calibration and constant comparison, independent of prior theoretical expectations. This literature provides essential perspectives for shaping codes that reflect the complexity and contemporary relevance of these subjects (Table S1, column 2, Appendix S1). For example, the literature on meat alternatives suggests that regulations on food safety and sustainability are becoming increasingly important in policies aimed at transitioning to sustainable food systems (Ritchie et al. 2022). Furthermore, Amato et al. (2023) underscore the complexity of transitioning to alternative meat from the perspective of stakeholders, who mediate between market demands and technological resources. In contrast, Fasolin et al. (2019) emphasize the importance of evaluating the safety of emerging proteins, suggesting a cautious approach to mitigate allergenic risks.

Studies on sustainability in the food sector highlight the need to integrate ethical and ecological practices to meet environmental and consumer demands (Foley et al. 2011). Codes related to technological innovation and cross‐sector collaboration are based on research that emphasizes the importance of partnerships among the public sector, private sector, and academic institutions in supporting sustainable innovation (Etzkowitz and Leydesdorff 2000). de Amstalden (2024) proposes an “open science” model in cellular agriculture to support accessibility and fair distribution of technological innovations.

Nguyen et al. (2022) highlight that factors influencing alternative protein consumption include opportunities and motivations. In contrast, Onwezen et al. (2021) demonstrate that the acceptance of meat alternatives depends on factors such as taste, health, and food neophobia. Mancini and Antonioli (2022) emphasize the importance of regulations and consumer education in the adoption of cultured meat, whereas Kwasny et al. (2022) propose a framework for reducing meat consumption, highlighting tailored interventions on the basis of social and behavioral variables. Tziva et al. (2020) indicated that enhancing both cognitive and normative legitimacy can drive the expansion of markets for sustainable products. Additionally, Bianchi et al. (2018) suggest that restructuring physical micro‐environments, such as reducing meat portion sizes, can support lower meat demand. In contrast, Graça et al. (2019) emphasize that motivation and opportunities play essential roles in shifting toward plant‐based diets. Van Loo et al. (2020) find a dominant preference for traditional meat, even in the presence of price reductions and well‐known brands for alternative products.

The study by DeMuth et al. (2023) finds that labeling restrictions on meat alternatives do not reduce consumer confusion, highlighting the complexity of consumer perception. Takeda et al. (2023) refer to the need for clear labeling regulations to support consumer trust, whereas Chiles (2013) explores how concepts such as “hype” and ideology have contributed to the evolution of in vitro meat, despite cultural and financial challenges. In addition, Petersen et al. (2021) suggest that front‐of‐package labeling does not necessarily ensure accurate perception of nutritional quality, whereas Brooker et al. (2022) show that alternative products are marketed as convenience foods with varied store placements, which influence consumer perceptions.

Moritz et al. (2024) discuss the importance of equity in the agricultural transition, warning that cellular agriculture could create regional inequalities if not handled correctly. Katz‐Rosene et al. (2023) describe three sustainability meta‐narratives for proteins (i.e., modernization, reconstitution, and regeneration), highlighting diverse but complementary approaches to creating more resilient food systems. Additionally, literature on animal welfare and ethical transitions suggests the need for standards that ensure respect for animal rights within an appropriate ethical framework (Fraser 2008). Moreover, de Boer and Aiking (2022) address aspects of animal welfare, emphasizing that reducing meat consumption can support a more sustainable diet. They focus on the “Reduce, Replace, and Refine” principles to lower environmental impact and improve animal health. Similarly, Chen et al. (2024) highlight that growing consumer awareness of animal welfare concerns is a key driver in the adoption of plant‐based alternatives such as plant‐based eggs, indicating a shift in values toward more ethically aligned food choices.

The role of media is also reflected in several deductive codes. Its importance is reflected in the literature on meat replacers. Hopkins (2015) considers that Western media present a biased view of the challenges associated with the acceptance of cultured meat, often overstating the role of vegetarian acceptance of this product. The study recommends that advocates of cultured meat focus on broader demographic insights, as strict vegetarians represent only a small segment of the population. Mroz and Painter (2023) reveal, on the basis of a content analysis of UK online news articles, a strong bias toward anti‐meat narratives, with most articles recommending reduced meat consumption and few supporting meat consumption or the meat industry, especially industrial farming practices. On the contrary, a study by Chen and Zhang (2022) that leverages social media data to analyze public attitudes toward alternative meat in China indicates mixed sentiments: fewer positive views on meat alternatives than previous studies suggested, and identifies unique factors influencing acceptance, such as conspiracy theories and a preference for traditional substitutes like tofu.

The selected deductive codes emphasize that transitioning to more sustainable food systems needs a cross‐sectoral approach, integrating regulation, innovation, education, and equity. This integration is crucial for addressing the complexity of consumer preferences, policy interventions, and regulations, as well as overcoming the challenges associated with the transition to alternative meat products.

### 2.4 Thematic Coding and Data Organization

After the interviews were recorded, they were transcribed, and thematic coding was applied using a structured coding guide derived from the research questions. Codes were iteratively reviewed by two researchers, with constant comparison (Corbin and Strauss 2014) to refine and merge overlapping ones and to ensure analytical rigor. Themes were then organized thematically, following the SPECT framework, which facilitated a coherent narrative of how Romanian decision‐makers perceive the opportunities, barriers, and policy implications of meat alternatives. The hybrid deductive‐inductive approach enabled theoretical grounding while preserving sensitivity to emergent categories that reflected local political realities. The process emphasized reflexivity and validation throughout, aligning with best practices in qualitative reliability (Nowell et al. 2017).

Although the SPECT framework provides a structured analytical lens, the empirical analysis revealed significant overlaps between dimensions. For instance, it is often difficult to clearly separate Policy and Regulation from Commercial Trends, as policies on labeling, subsidies, or food safety directly influence the commercial viability of alternative products. These intersections reflect the complexity of food transitions, justifying a flexible analytical approach that remains sensitive to the interdependencies between dimensions.

## 3 Results

**FIGURE 2:**
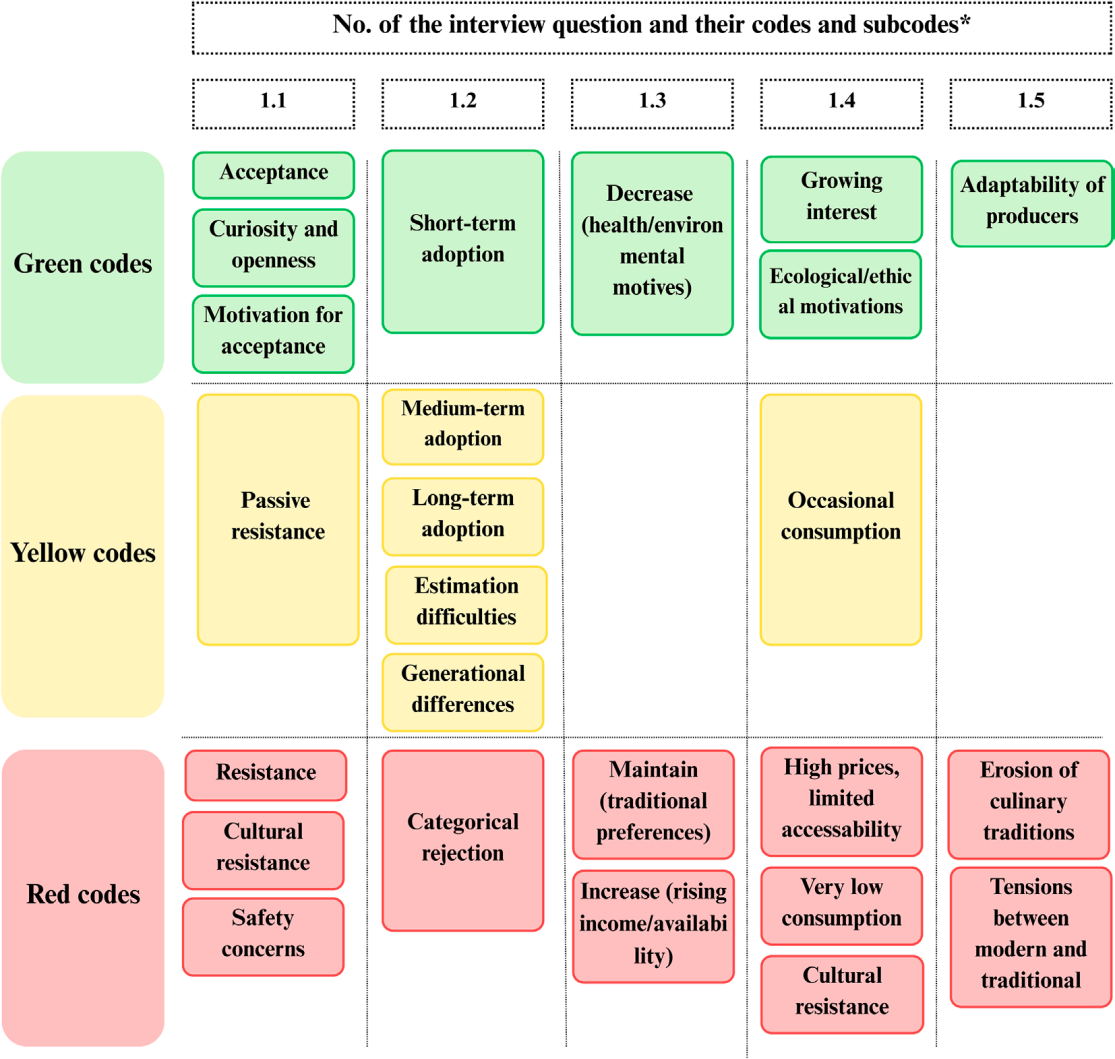
Social and cultural acceptability in traffic light visualization (yellow: Neutral attitude; green: Openness; red: Resistance). *The interview questions are available in Table S1, Appendix S1.

**FIGURE 3:**
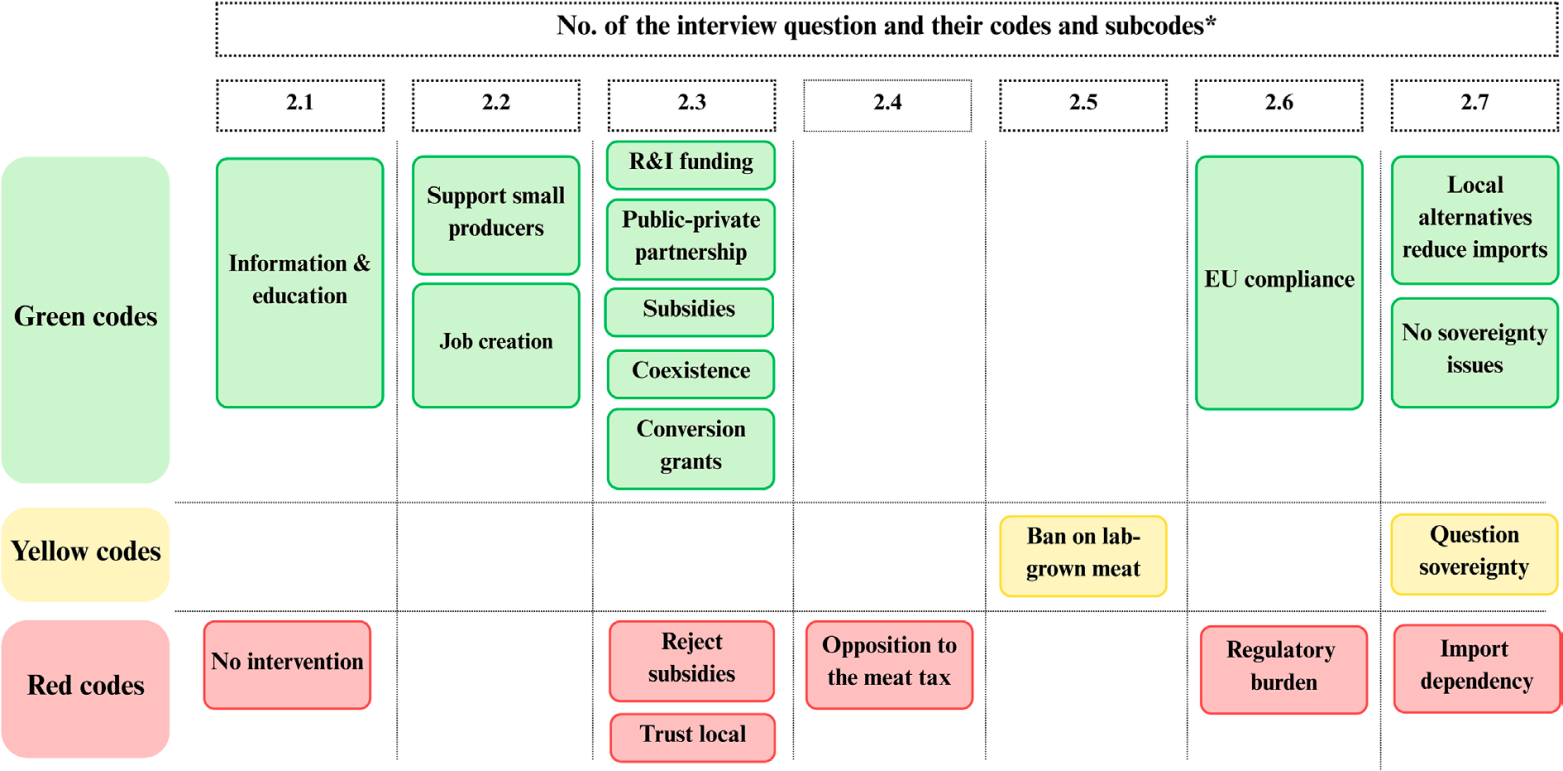
Policy and regulation dimension in traffic light visualization (yellow: middle‐ground perspectives; green: support; red: resistance). *The interview questions are available in Table S1, Appendix S1.

**FIGURE 4:**
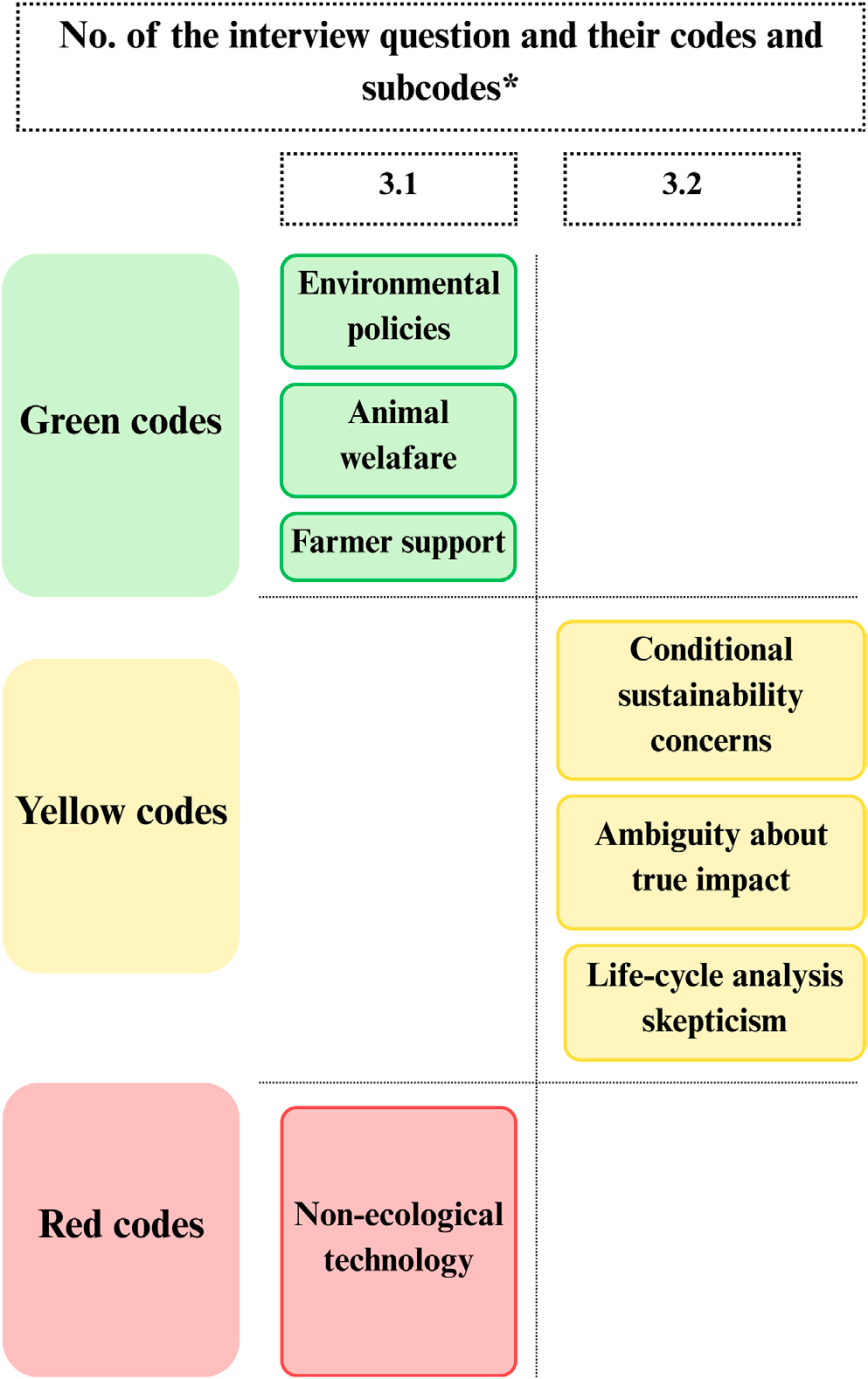
Environmental impact and animal welfare dimension in traffic light visualization (yellow: Ambivalence; green: Alignment with sustainability principles; red: Resistance). *The interview questions are available in Table S1, Appendix S1.

**FIGURE 5:**
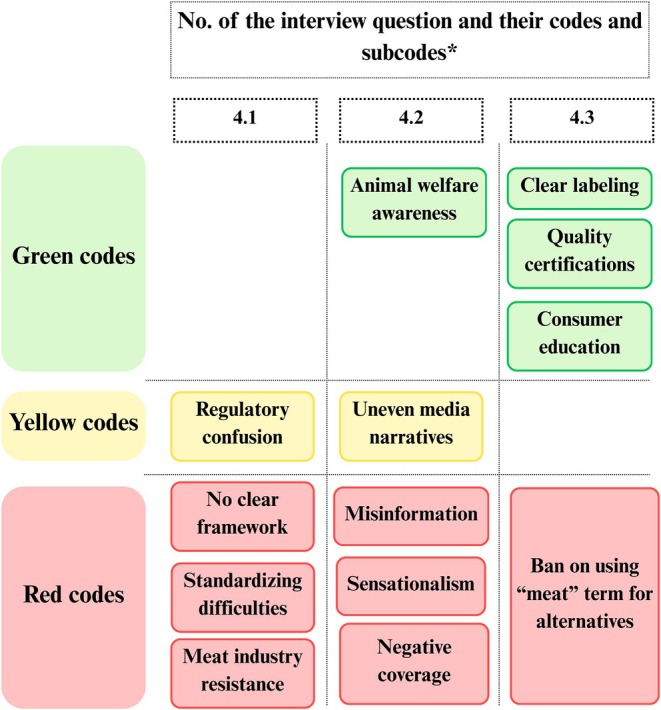
Commercial and communication in traffic light visualization (yellow: Ambivalence; green: Support; red: Barriers). *The interview questions are available in Table S1, Appendix S1.

**FIGURE 6:**
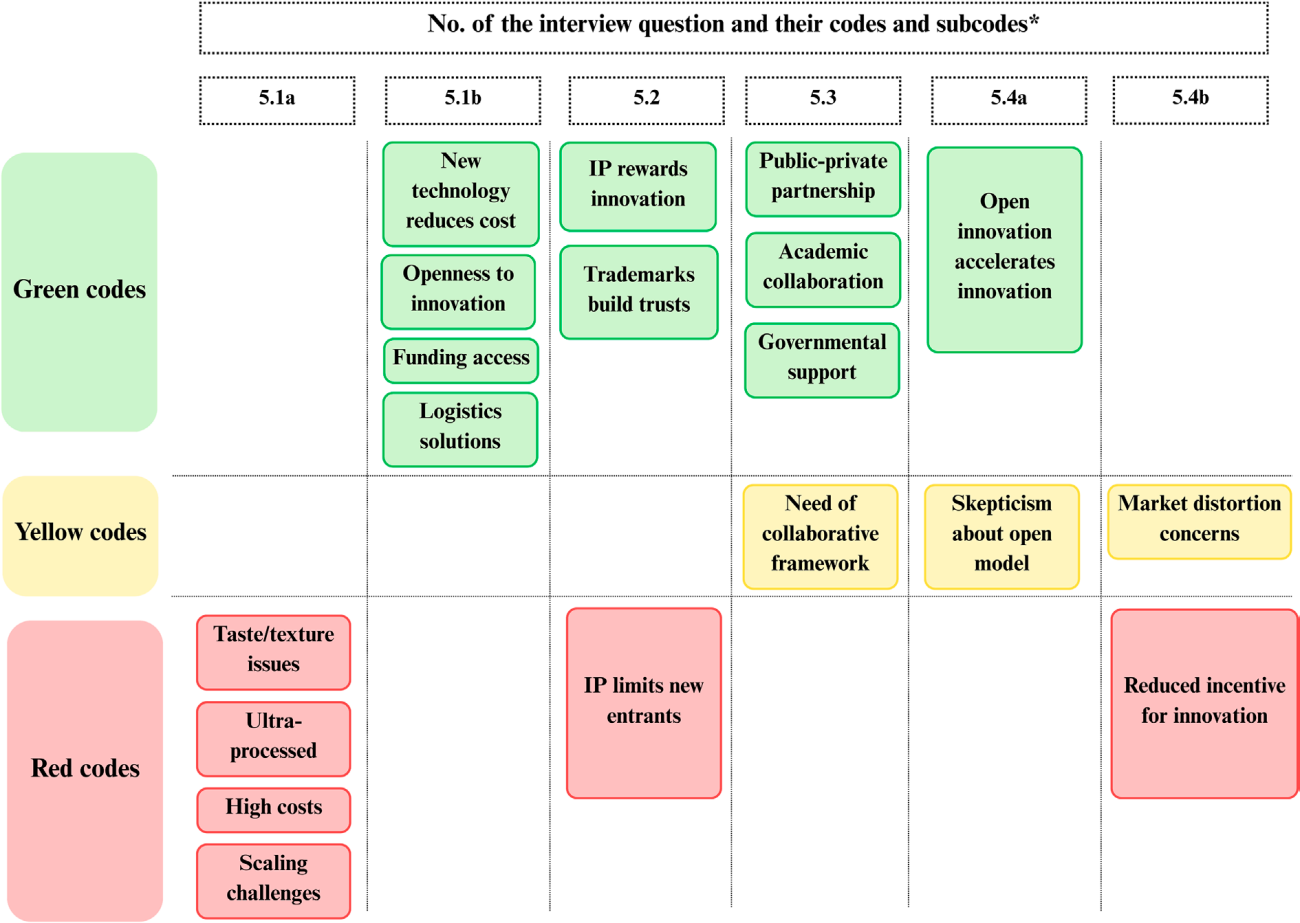
Technological development and innovation in traffic light visualization (yellow: Complications; green: Optimism; red: Limitations). *The interview questions are available in Table S1, Appendix S1.

The key themes that emerged across each dimension of the SPECT framework are presented using five traffic light figures (Figures 2, 3, 4, 5, 6). Each figure groups the main codes and subcodes by interview question and categorizes them using the traffic light colors. Green was used for supportive or enabling views, yellow for ambivalent or conditional perspectives, and red for resistant or negative ones. This visual synthesis allows for quick comparison of patterns across social, political, environmental, commercial, and technological domains.

Although each figure highlights the overarching narrative, detailed explanations of each code, subcode, and examples of supporting participant quotes are available in the Appendix S1. Representative quotations were integrated here to illustrate key findings and strengthen the empirical connection between participants' voices and thematic claims. Together, the quotes, figures, and the Appendix S1 offer a high‐level summary and an in‐depth understanding, respectively, of participants' perceptions of meat alternatives. Terms such as “strong culinary conservatism” and “hostility toward the EU” are used as analytical categories derived from recurring patterns in the data and are kept conceptually distinct from the empirical quotations, which are reported descriptively. Regarding participant quotations, we include in the main text the most representative voices, whereas an extensive reference to all quotes is provided in Table S1 (Annex).

The green codes and subcodes in the *Social and cultural acceptability dimension* (Figure 2) (e.g., acceptance, curiosity, and openness) indicate positive or supportive views toward meat alternatives. Several participants, often younger or urban, expressed curiosity and openness, citing health and sustainability as key motivations. As P1 observed, “urban residents are more open‐minded than those in rural areas,” whereas P9 added that “these alternative products may be healthier for the human body than conventional meat.” Others shared similar sentiments, for example, P6 explained that “people want to eat healthy, and they will start buying what they perceive as better for them.”

Yellow codes (e.g., passive resistance, occasional consumption) reflect neutral or ambivalent views, often tied to limited awareness or exposure. P17, also involved in animal husbandry, said “We don't really know what alternative meats contains […] We don't know the effects, and we don't know how it's produced. That's exactly where my skepticism about this type of meat comes from.” Some admitted being uninformed and thus neither supportive nor opposed, whereas others expected adoption only in the distant future. P6 said, “I believe that the vast majority are like me, uninformed in this area… and I think they are reluctant, considering our culture and way of life.” Red codes (e.g., resistance, cultural resistance, safety concerns) highlight views that are rejected. Resistance, particularly toward insect‐based products or cultured meat, was described as cultural. P2 emphasized that “Romanians are traditionalists. They like homemade food, the borscht from Moldova, and so on.” Others expressed disgust toward insect‐based or cultured meat foods, and some voiced distrust in their safety. P8 argued that “I would never eat something made in a lab; I don't know what's inside it.” Overall, the social context reveals patterns coded as strong culinary conservatism and knowledge gaps that are associated with resistance (red), with openness (green) primarily among informed political and institutional actors. Neutral attitudes (yellow) arise from caution or lack of familiarity.

In the *Policy and regulation* SPECT framework, green codes reflect supportive policy perspectives. Many participants endorsed informational campaigns and education to reduce cultural aversion. P5 suggested that “people need to understand why these products are necessary,” whereas P3 emphasized that “public‐private partnerships are crucial to developing this market.” Participants also valued state incentives, with P6 noting that “rural producers will need financial support and training to adapt.” Likewise, P16 considered that “Trainings, educational programs, and experience exchanges could help make this transition smoother.” P15 repetitively mentioned the role of “transition funds” for conventional meat producers. Yellow codes show conditional or nuanced views. Some acknowledged that globalization limits national autonomy but recognized Romania's dependence on EU policy frameworks. P10 remarked, “Romania's food independence is always partial; we're part of global markets.”

Red codes capture resistant or protectionist attitudes. Some participants opposed government intervention, arguing that there is “no need for policies to reduce cultural resistance” (P9), as traditional food choices should be respected. Most participants strongly rejected a “meat tax”, with P4 warning it would “hurt small producers and burden consumers.” Support for banning cultured meat was often driven by skepticism and nationalism. P2 called the proposal “a very good initiative” to “protect people from unnatural food,” whereas P8 echoed that such meat “shouldn't even be allowed to exist.” These positions reflect a desire to preserve conventional agriculture and culinary heritage. A few also distrusted EU regulations, viewing them as externally imposed and damaging to food sovereignty. For example, P8 argued, “They're pushing these rules on us as if we were living in Africa,” a statement coded under hostility toward the EU. Although many saw no threat to sovereignty (P1, P7), one dissenting view (P8) held that international laws “severely affect our ability to choose.” Overall, political opinions ranged from proactive support (green) to firm resistance (red), with some middle‐ground perspectives (yellow) focused on structural realities (Figure 3).

In the *Environmental impact and animal welfare* dimension of the SPECT framework (Figure 4), green‐coded responses reflect strong alignment with sustainability principles. Many endorsed policy and legislative actions to reduce the ecological impact of food production. However, this strong normative support for sustainability frequently coexists with rejection of meat alternatives as concrete transition pathways. This reveals a form of cognitive dissonance, whereby participants endorse sustainability as a principle while simultaneously resisting the very innovations proposed to operationalize it. Several pointed to Romania's alignment with EU goals. Thus, P1 noted that “40% of agricultural funds now focus on the environment,” which signals approval of such measures. Strong support also emerged for animal welfare improvements, with P7 stating animals “must live in decent, hygienic conditions until that moment” of slaughter, even if traditional farming continues. Another green theme was supporting farmers in adopting sustainable practices. P16 says, for example, that “conventional animal farming requires significantly more, and more expensive, resources, starting with feed and water, which are becoming increasingly costly and scarce.” Participants favored allocating funding or resources to support small producers' transition, viewing it as mutually beneficial for both rural livelihoods and environmental goals.

Yellow codes captured ambivalence. Some participants acknowledged potential benefits but raised questions about life‐cycle impacts, resource use, or long‐term consequences. For example, P10 said, “It depends on how they're produced, if they need more energy or water, then it's not really sustainable.” These views neither embraced nor rejected meat alternatives outright; rather, they called for more data and transparency before forming a clear opinion.

The red subcode code “alternative protein technologies seen as non‐ecological” captures opposing views. Few participants doubted that cultured meat or insect‐based products are environmentally sound. P8 argued they “seriously harm biodiversity” and cause a “major environmental impact,” rejecting claims of sustainability. These critics saw such technologies as artificial (“parallel worlds” to real ecology) and expressed mistrust of industrialized innovations.

In the *Commercial and communication* dimension, participants emphasized a mix of red‐coded barriers and green‐coded opportunities. Red‐coded issues predominated, particularly in areas related to regulation and industry competition (Figure 5). P10 explained, “Each product, synthetic, insect, or plant‐based, has different regulations, and there is no common standard yet.” P3 warned, “If these alternative products grow, tensions will surely appear.” At the same time, P17 confessed, “Creating something new, which is actually the opposite of our work, feels like an enemy.” These statements illustrate the commercial friction between emerging and conventional sectors. Media coverage was also viewed negatively. Participants agreed that Romanian media often sensationalize or distort information on alternative meat. P1 noted having “only heard negative reactions,” whereas P5 remarked, “people might be more drawn to spectacle… than to actual information.” This focus on shock over facts fosters public distrust. As P4 and P11 added, the media “didn't seem particularly fair” and “contributes to misinformation,” suggesting that both mass and social media hinder informed consumer choices, justifying red codes. Still, one positive statement was made by P18: “Because of media coverage of issues related to animal welfare and sustainability, people have become more aware of animal farming practices on small plots of land with high density, their transport, and especially their slaughter. For example, the local slaughter points that used to operate during Easter fairs, where lambs.”

Green codes revealed constructive ideas: labeling, certification, and communication strategies. P1 proposed that “labels should be clear and distinct, just like organic products,” and P18 added that “because of media coverage of animal welfare and sustainability, people have become more aware of the need for humane farming.” Education campaigns were widely supported to explain these products and how to interpret labels. Although some doubted their full effectiveness because of media mistrust, the principle of public education was widely regarded as beneficial.

Notably, a few views were more cautious than rejecting, and they were presented in yellow codes. Some expressed confusion about who should regulate these products (P6), reflecting uncertainty rather than outright opposition. Others viewed media coverage as mixed, neither entirely negative nor helpful, which led to varied interpretations of the landscape of alternative meat products.

One red‐coded issue involved the proposed ban on using the word “meat” for alternative products. A few participants argued against allowing such terminology. P8 stated producers “don't have the moral… right to call cultured meat ‘meat’,” considering it misleading. This stance reflects a desire to protect conventional meat product categories and signals resistance to normalizing alternatives under familiar labels. Overall, participants identified significant commercial hurdles, including legal ambiguity, industry pushbacks, and biased media, which were highlighted as red‐coded barriers. At the same time, decision‐makers and institutional actors proposed green‐coded strategies such as transparent labeling, third‐party certification, and consumer education to foster trust and readiness for meat alternatives. The presence of yellow‐coded views adds nuance, capturing ambivalence and the need for clarity in both regulation and public messaging (Figure 5).

In the *Technological development and innovation* dimension, participants identified a mix of significant hurdles (red), promising drivers (green), and ambivalent or conditional factors (yellow). Red‐coded barriers centered on product quality, health perceptions, and cost. A major concern was that meat alternatives still fail to replicate the sensory experience of conventional meat. P5 noted, “Current tech and infrastructure do not yet provide the greatest benefits when it comes to imitating conventional meat… in texture and taste.” Participants also raised concerns about the “ultra‐processed” nature of meat alternatives, linking additives and artificial ingredients to consumers' hesitation. P5 explained that “(…) the current technology and infrastructure do not yet provide the greatest benefits when it comes to imitating conventional meat, in terms of texture and taste, and that makes consumers reluctant. Also, as I mentioned, these products tend to be ultra‐processed. P5 added that this perception is ‘the biggest challenge… to overcome through technology’,” since many consumers distrust heavily processed foods. High production costs and limited scalability were also identified as major barriers. Several mentioned that cultured meat requires expensive infrastructure and remains risky. As P3 put it, “There are social, legislative, and economic barriers… producers might lack the courage to adopt these technologies in the short term.” These limitations, technical, economic, and perceptual, explain the dominance of red codes in early‐stage meat alternatives technology.

Green‐coded reflected optimism about innovation. Participants highlighted that new tech could reduce costs and improve quality over time. There was also a strong belief in cross‐sector collaboration. P1 and P3 emphasized the importance of public–private partnerships and academic cooperation in driving progress. P3 stated, “Only collaboration between sectors will put these products on shelves.” Many valued Romania's existing agricultural innovation networks, with P1 referring to AKIS (Agricultural Knowledge and Innovation System) as a model for knowledge exchange. Participants noted openness to innovation in Romania's agri‐food sector, with P1 pointing to farmers using “robots and drones” as a foundation for future technology adoption. Funding was also seen as critical; European or government support could stimulate R&D and infrastructure development. Logistical improvements (e.g., cold chains) were noted as practical enablers of wider distribution.

Yellow codes emerged in more nuanced discussions. Although collaboration was broadly welcomed, P5 emphasized the need for a formal framework: “We must have organized platforms; otherwise, it won't happen.” Similarly, attitudes toward open‐source models were mixed. Some appreciated the idea of speeding up innovation, but others were skeptical, questioning whether companies would still invest in R&D if profits weren't secured. This ambivalence was coded yellow, especially considering concerns over sustainability and long‐term access.

Intellectual property (IP) also sparked both red and green views. On the one hand, P1 and P3 defended IP as essential for innovation, as it rewards “true pioneers” and supports branding and consumer trust. On the other hand, critics revealed it could block access. P2 strongly opposed patents, claiming they enable corporations to “destroy…health,” whereas P13 stated that patents mean “no one else can use the product.” These quotes reflect tension between protecting innovation and ensuring open access.

Overall, participants recognized significant technological barriers but expressed hope that targeted innovation, funding, and structured collaboration could transform the sector. Green codes reflected this optimism, red codes warned of real present limitations, and yellow codes acknowledged conditions or concerns that complicate a linear path forward (Figure 6).

## 4 Discussion

### 4.1 Reframing the Transition to Sustainable Meat Alternatives: Political and Institutional Actors as Emergent Stakeholders

This study reveals that political and institutional actors, often viewed as policy implementers rather than opinion shapers, are, in fact, influential co‐authors of sustainable food transitions. Their perceptions actively construct or constrain legitimacy, shaping public narratives, regulatory priorities, and the pace of innovation. Importantly, this characterization should be understood as interpretative analytical framing derived from patterns in the qualitative data, rather than as a direct empirical claim about institutional agency or formal power. The notion of “co‐authorship” is therefore used heuristically to capture how interviewees discursively position themselves in relation to the transition, without implying institutional representativeness or normative endorsement. This finding adds new empirical depth to scholarship that has traditionally focused on consumer acceptance (Onwezen et al. 2021), technological innovation (Chriki et al. 2022), or entrepreneurial dynamics (Amato et al. 2023). By foregrounding how these actors negotiate between national identity, policy coherence, and trust in innovation, this study exposes an underexplored layer of sociopolitical mediation within protein transitions.

#### 4.1.1 *Social Acceptability (S)*: Cultural Inertia vs Emerging Curiosity

The findings reveal a deep cultural attachment to traditional meat consumption among Romanian political and institutional actors, particularly pork and home‐raised livestock. Plant‐based products, however, were often perceived as more familiar and adaptable, with several participants describing them as “palatable bridges” between tradition and innovation that could mediate between heritage and sustainability. Participants' statements indicated that food practices are embedded in moral and identity frameworks rather than merely driven by nutritional considerations. As Caplan (2021) notes, food reflects belonging and moral order. In this study, conventional meat was perceived as symbolizing authenticity and trust in “real food,” whereas cultured meat and insect‐based products elicited disgust and skepticism. Yet signs of change appeared among younger or progressive actors, who expressed curiosity and saw education as key to acceptance, echoing Szenderak et al. (2022) and White et al. (2022), who argue that shifting perceptions are possible through targeted education and cultural adaptation. The findings suggest that in the Romanian context, plant‐based proteins may act as a transitional bridge, being perceived as less “unnatural” than cultured meat or insect options. This dynamic aligns with the concept of “culinary scaffolding” (Van der Weele et al. 2019), where meat alternatives succeed not by perfectly mimicking meat, but by integrating into familiar practices. This dynamic highlights a generational divide where openness (green‐coded) coexists with caution (yellow) and cultural resistance (red). Political actors can facilitate this scaffolding through strategic public messaging and policy incentives.

#### 4.1.2 *Policy and Regulation (P)*: Polarized Ideologies and Policy Inconsistency

A defining feature of Romania's policy landscape is its ideological polarization. Supportive actors advocate innovation through public–private partnerships, transition funds, and R&D support, whereas protectionist voices reject cultured meat and invoke sovereignty narratives. This tension reflects two competing ideological projects—eco‐modernization versus agrarian nationalism—each shaping the interpretation of the concept of “sustainability” in policy discourse. These dualities echo with broader European trends. As noted by Hundscheid et al. (2022), sustainable protein transitions often suffer from policy incoherence and institutional inertia. When governments promote meat reduction rhetorically while simultaneously subsidizing conventional livestock production, stakeholders receive mixed signals, thereby delaying progress. In Romania, this incoherence is intensified by nationalist rhetoric that frames EU regulation as a foreign imposition. Such framings transform food transitions into ideological battlegrounds rather than merely regulatory debates. This reflects global patterns in which food transitions are often interpreted as geopolitical struggles rather than as public health or environmental imperatives (Nützenadel and Trentmann 2008). Political actors thus function not only as policy designers but as narrative custodians whose values shape public legitimacy.

#### 4.1.3 *Environmental and Animal Welfare (E)*: High Awareness, Limited Integration

Participants widely acknowledged the urgency of climate change and biodiversity challenges but displayed a compliance‐based rather than visionary orientation. Environmental goals were often discussed as obligations under the EU Green Deal rather than as proactive national strategies. Although several actors supported improving animal welfare and sustainable farming, few connected these priorities to the development of meat alternatives. This gap mirrors European debates (Hocquette et al. 2025) where the ethical promise of cultured meat and insect‐based food depends on public trust and local innovation capacity. As shown by Chriki et al. (2022), the sustainability of cultured meat and insect‐based products depends on scale, energy sources, and public trust. Without national R&D investment, Romanian political and institutional actors perceive these alternatives as external and industrial rather than local and ethical.

#### 4.1.4 *Commercial and Communication (C)*: The Missing Infrastructure of Trust

Another critical gap identified is the lack of coordinated public communication. Participants called for educational campaigns, more transparent labeling, and more accessible price points. These findings are echoed by Brooker et al. (2022), who argue that commercial success for novel proteins depends not only on product quality but on store placement, naming conventions, and narrative framing. Yet misinformation and sensationalist media coverage, often invoking terms like “synthetic” or “unnatural,” reinforce fear and moral panic. These results align with those of Chen and Zhang (2022), who note that polarized narratives around alternative proteins invite ideological resistance.

In Romania, where culinary heritage is closely tied to national identity, alternative meat products are often portrayed as foreign, artificial, or threatening (Petrescu‐Mag, Petrescu‐Mag, et al. 2025). Importantly, participants called for communication strategies that connect innovation to national identity rather than oppose it. Avoiding “eco‐elitist” tones and framing change as culinary continuity were seen as ways to bridge skepticism. Political and institutional actors thus emerge as mediators of meaning, that is, positioned between scientific knowledge and societal trust. Conceptually, this study advances the notion of political and institutional actors as mediators of meaning, defined as actors who do not merely regulate or implement policy, but actively translate, frame, and negotiate the symbolic boundaries of sustainability between scientific expertise, national identity, and public trust. This positions them as central interpretive agents in food system transitions, rather than as neutral governance intermediaries. In this context, political and institutional actors have a unique opportunity to serve as narrative translators, mediating between scientific innovation and societal values.

#### 4.1.5 *Technology and Innovation (T)*: Underdeveloped Ecosystems, Overreliance on Imports

Participants agreed that Romania lacks an innovation ecosystem for alt‐proteins. Research institutions are underfunded, and public–private collaboration remains weak. Many actors perceived novel proteins, especially cultured meat, as “imported projects”, detached from Romania's agricultural tradition, an attitude that reinforces skepticism and dependency concerns. This perceived disconnection reinforces skepticism and fuels concerns about dependency on foreign technologies. Strengthening locally grounded innovation pathways is therefore essential to enhance legitimacy and reduce resistance. Unlike countries with established academic‐industry partnerships, such as the Netherlands or Israel, Romania's research infrastructure remains fragmented. As highlighted by Hocquette et al. (2025), a successful protein transition depends not only on technical breakthroughs but also on coordinated regulatory, ethical, and financial infrastructures.

### 4.2 A Brief Overview of Participants' Opinions Through Ideological Filters and Practical Implications

Without developing domestic production capacity, Romania risks remaining a passive importer of meat alternatives, which could reinforce nationalist backlash and deepen perceptions of dependency. Building domestic capacity requires targeted support: grants for plant protein processing, university R&D in cellular agriculture, and support for hybrid (traditional and alternative) models that leverage local culinary traditions. These practical needs are deeply intertwined with the ideological filters through which political actors interpret the protein transition.

Although the study did not aim to measure political ideologies directly, clear patterns emerged across party lines, revealing how worldviews shape openness or resistance to food innovation. Participants affiliated with far‐right or nationalist parties [e.g., (P8)] were among the most vocal opponents of meat alternatives, particularly cultured meat, which they associated with unnaturalness, cultural erosion, or even environmental harm. P2 supported the ban on cultured meat as a necessary step to “protect the Romanian people from synthetic food.” At the same time, P8 argued that cultured meat “harms biodiversity” and represents “an artificial substitute with political goals.” These narratives exemplify what Nützenadel and Trentmann (2008) refer to as the moral economies of food, where national sovereignty and authenticity prevail over environmental or technological considerations.

By contrast, center‐left and green‐progressive actors [e.g., (P3), (P4), (P10)] supported proactive measures such as educational campaigns, transition funds, and collaborative policy design. Their views emphasized health, climate, and social equity arguments, aligning with EU sustainability discourses (Hundscheid et al. 2022). Center‐right and centrist actors [e.g., (P6), (P7), (P9), (P11)] occupied a pragmatic middle ground, recognizing the potential of meat alternatives but warning against disruptive regulatory shifts. They favored gradual integration through fiscal incentives and innovation‐oriented subsidies rather than bans or mandates. Members from one party from this category showed more heterogeneous views, ranging from openness to innovation to firm cultural conservatism. Although not conclusive, these patterns suggest that actors approach the transition to meat alternatives through distinct ideological filters that shape their views on legitimacy, risk, and national priorities. It is worth reiterating that the opinions expressed by participants affiliated with public institutions or political parties are solely their own and do not reflect the official positions of the institutions or parties they are associated with (as mentioned in Table 1).

Together, these ideological gradients (from protectionist to pragmatic to progressive) shape how legitimacy, risk, and responsibility are constructed across Romania's political spectrum. However, these patterns should be interpreted as exploratory and indicative, reflecting the situated perceptions of a limited set of interviewees rather than institutionally representative positions of political parties or the Romanian political system as a whole. Recognizing these ideological undercurrents is essential for designing effective policy strategies that can gain multi‐partisan support and public legitimacy. To move from diagnosis to action, it is important to consider the *broader practical implications and limitations of this research*. From a practical standpoint, this study highlights several priorities:

- Reposition political and institutional actors as proactive stakeholders in food innovation ecosystems, rather than treating them merely as regulators or opponents.
- Develop communication and educational strategies that reflect Romanian cultural norms, emphasizing culinary continuity rather than rupture.
- Integrate traditional producers into new value chains through training, hybrid product development, and public–private partnerships to foster inclusion and rural resilience.
- Invest in domestic research infrastructure to strengthen national food sovereignty and reduce dependency on foreign innovation.

By linking ideological insights with structural recommendations, the study demonstrates that trust and cultural alignment are as crucial to a just protein transition as technological advancements and that achieving national food sovereignty requires context‐specific solutions.

The findings of this study should be interpreted in light of several conceptual and methodological limitations, which also open avenues for future research on just protein transitions. Although the SPECT framework proved useful for structuring institutional perceptions, it does not fully capture deeper power relations, geopolitical dynamics, or global political economy factors (e.g., corporate concentration, trade regimes, and lobbying) that also shape protein transitions. Future research could therefore complement SPECT with political economy or multi‐level governance frameworks to better account for structural inequalities and transnational influences beyond the national institutional level. Also, the relatively small, purposefully selected sample limits the generalizability of the findings, especially given the sensitivity of the topic and the institutional positions of some participants. Because recruitment partly relied on political networks and personal contacts, selection bias may have occurred, favoring participants who were more open to discussion or embedded within specific party environments. Snowball sampling may also have skewed the sample toward more connected or ideologically aligned individuals, potentially excluding dissenting voices from marginalized or rural institutions. However, this limitation was mitigated through purposive diversification across political affiliations and institutional types (Palinkas et al. 2015; Patton 2002) and by cross‐validation of interview insights to strengthen representational breadth. The qualitative nature of the study provides depth, but not breadth, and should therefore be interpreted as exploratory groundwork for future empirical research. Expanding this stakeholder mapping through quantitative surveys or multi‐country comparisons would deepen the understanding of how governance cultures shape sustainability transitions in the food sector and significantly enhance the policy relevance of these findings. Future research should extend this analysis by examining levels of awareness, acceptance, and trust in alternative proteins among the general Romanian population to compare elite discourses with societal attitudes. Moreover, comparative studies across other Eastern European countries would enhance regional understanding and allow for a systematic assessment of how governance cultures shape protein transition pathways.

## 5 Conclusion

The study explored pathways to a just transition in Romania's meat alternatives sector from the perspectives of political and institutional actors, a group often overlooked in food systems research. Using the SPECT framework, we examined how these actors perceive and position meat alternatives across five key dimensions: social acceptability, policy and regulation, environmental impact, commercial dynamics, and technological readiness. Although some respondents supported gradual integration through education and innovation, others expressed deep resistance rooted in cultural nationalism, skepticism of technology, or distrust of EU governance. The findings reveal a landscape of contested narratives and competing priorities, where sustainability goals are filtered through ideologies, traditions, and institutional constraints.

The findings demonstrate that these actors are not neutral mediators but value‐laden stakeholders whose influence extends far beyond regulation. Their perceptions shape public discourse, legislative action, and the strategic framing of new food technologies. Achieving a just and culturally sensitive protein transition will require more than technical solutions. It demands inclusive governance, targeted public engagement, and capacity‐building within domestic research ecosystems.

Beyond its empirical contributions, this study also offers a clear theoretical advance for the literature on just transitions and sustainable protein governance. Theoretically, this study extends just transition scholarship by shifting the analytical focus from citizens, firms, or technologies to institutional sense‐making, showing that political and institutional actors function as interpretive gatekeepers who actively shape the legitimacy, narrative framing, and governance pathways of protein transitions. In doing so, it nuances existing governance models by demonstrating that transitions are mediated not only by policy instruments, but by culturally embedded meanings of sovereignty, trust, and national identity. These theoretical insights have direct implications for policy design, suggesting that effective interventions must go beyond technical regulation and economic incentives to also address institutional narratives, legitimacy‐building, and cultural alignment. From a policy perspective, several practical directions emerge. First, Romania could introduce fiscal incentives, such as tax credits or grants, for small and medium producers investing in plant‐based or hybrid protein lines. Second, integrating alternative protein topics into the National Rural Development Programme and vocational training curricula could help farmers diversify production without losing income stability. Third, establishing a public–private “Protein Innovation Hub” under the Ministry of Agriculture would strengthen coordination between research institutions, food companies, and policymakers. Fourth, national labeling and communication campaigns that emphasize transparency, cultural continuity, and food safety could help build consumer trust and counter misinformation. Finally, redirecting a portion of agri‐environmental funds toward low‐emission protein crops (e.g., peas, soy, lupin) would align Romania's agricultural strategy with EU Green Deal objectives while maintaining local sovereignty. These targeted actions, focusing on financial support, education, innovation infrastructure, and narrative alignment, translate the conceptual goals of inclusive innovation and trust‐building into feasible policy mechanisms for Romania's current institutional landscape.

## Author Contributions

**Ruxandra Malina Petrescu‐Mag:** conceptualization, data curation, formal analysis, funding acquisition, investigation, methodology, project administration, supervision, validation, visualization, writing – original draft, and writing – review and editing. **Camelia Ginsca:** data collection, validation, and visualization. **Ioana Pistea:** data collection. **Kinga Olga Reti:** data collection and validation. **Cristina Bianca Pocol:** writing – original draft. **Radu Cristian Maricut:** data collection. **Dacinia Crina Petrescu:** conceptualization, data curation, formal analysis, methodology, validation, writing – original draft, and writing – review and editing.

## Funding

This work was supported by the Ministry of Education and Research, Romania, ERANET‐JPI‐HDHL‐ComMEATted.

## Ethics Statement

This study does not involve any human or animal testing. The research received Ethical approval no 7151/24.05.2024 from Babes‐Bolyai University, Cluj‐Napoca, Romania.

## Consent

Written informed consent was obtained from all study participants.

## Conflicts of Interest

The authors declare no conflicts of interest.

## Supporting information


**Appendix S1:** fsn371650‐sup‐0001‐AppendixS1.docx.

## Data Availability

The data that support the findings of this study are available from the corresponding author upon reasonable request.
