# Supplementary material for: Rethinking Meat Alternatives in Eastern Europe: A Just Transition Lens on Policy, Perception, and Innovation in Romania
Source: Food Sci Nutr. 2026 Apr 24;14(4):e71650. doi: 10.1002/fsn3.71650 (PMC13107119; doi:10.1002/fsn3.71650)
Supplement: Supplementary file 1 — Appendix S1: fsn371650‐sup‐0001‐AppendixS1.docx. [file FSN3-14-e71650-s001.docx]

**Appendix S1**

Table S1. A priori (codebook-based, deductive) and emergent (data-driven, inductive) codes with participants’ quotes

| **Interview questions** | **Code** | **Subcode** | **Explanation** | **Participants’ quotes (examples)** | **Inductive codes emerged from participants’ interviews** |
| --- | --- | --- | --- | --- | --- |
| ***SPECTA framework elements and supporting literature for deductive codes***  **s: Social and cultural acceptability**  (Bianchi et al., 2018; de Boer & Aiking, 2022; Foley et al., 2011; Graça et al., 2019; Mancini & Antonioli, 2022; Nguyen et al., 2022; Onwezen et al., 2021) | | | | | |
| RQ1. **What are the social and cultural factors influencing the acceptability of alternative meat products (cultured meat, insect-based food products, and plant-based food products) in Romania?** | | | | | |
| 1.1 What do you think is the **public’s attitude** (position) regarding these alternative meat products? | **Acceptance** | Curiosity and openness | Openness to exploring alternative meat products due to curiosity or other factors. | “I believe that, in general, **urban residents** are more open-minded than those in **rural areas**. This is probably related to the fact that in rural areas, regardless of the country, not just in Romania, people tend to be more **conservative**.” P1; “But if we’re talking about a student who moves to a big city, then yes – there, they tend to try new things. You know how students are: they don’t cook because they don’t have the space, and they’re more open-minded. When they see meat or prepared food, they look at what it’s made of, and they might give it a try.” P17; “in rural areas, people seem to be more reluctant, while in urban areas, they tend to be more open to such products.” P18►  “I think the discussion is still at an early stage, because these products have not been promoted very much. As a result, there is also a **lack of knowledge** about the products themselves.” P4 ► | ►* **Urban–rural divide in acceptance:** differences in perception and openness between the urban population (more open) and the rural population (more conservative) toward alternative meat products.  ► **Lack of knowledge**: regarding alternative meat products, which contributes to public hesitation or misunderstanding. |
|  |  | Motivations for acceptance | Motivations include sustainability, health, ethics, etc. | “These alternative products may be healthier for the human body than conventional meat, which in excess can cause health problems.” (P9)  “Yes, it could be said that they are healthier, especially the plant-based ones, because they do not contain cholesterol, they do not have excessive saturated fats, and they can help achieve a more balanced diet.” (P4)  “There are opportunities to overcome these problems related to health and environmental protection.” (P5)  “First of all, people want, if we look around, they want to eat healthy; the media communicates a lot of information about healthy living, healthy eating, replacing meat with plant-based products like soy or lentils.” (P6) |  |
|  | **Resistance** | Cultural resistance | Rejection of alternative meat products due to cultural beliefs or practices. | „ In **rural areas**, regardless of the country, not just in Romania, people tend to be more c**onservative**.” (P1)►  “From a gastronomic point of view, Romanians are traditionalists. They like traditional food, they like food from Maramureș, they like homemade food, the borscht from Moldova, and so on.” (P2)  “People had quite a negative perception. I believe that if the benefits were explained better, then the perception would change.” (P4)  “I think there is a certain reluctance. It is true that many times meat alternatives do not successfully imitate the taste and texture of conventional meat. And so, of course, there is this reluctance.” (P5)  “The general perception of people is negative because they do not fully understand what it is about.” (P11)  “In Romania, the population is reluctant when it comes to cultured meat products or meat obtained from insect processing, but in the case of plant-based products, I do not think there is reluctance.” (P12)  “I think Romanians have a rather limited mindset toward these products, and there is a big stigma toward countries that consume insects or insect-based foods.” (P7) | ► **Urban–rural divide in acceptance**: differences in perception and openness between the urban population (more open) and the rural population (more conservative). |
|  |  | Passive resistance | Public has no strong opinion on alternative meat products. | “I believe that the vast majority are like me, uninformed in this area, and I think that if they are not properly informed, they cannot express any opinion. And I think they are reluctant, at least in Romania, considering our culture and our way of life, like raising pigs in the household.” (P6)  “I think society is quite divided on this matter, but primarily because of misinformation (…) people **do not really understand** what this is about and are quite resistant to this change.” (P7) ►  “This is not a topic currently present in the public space, so the public doesn’t have a clear point of view. It is still in the domain of professional studies and less in the public sphere.” (P8)  “At this point, I think the attitude is one of caution, just like mine. If I don’t know, haven’t tasted it, I don’t have a negative or positive opinion – it’s neutral.” (P9)  “The **general perception of people is negative** because they do not fully understand what it is about.” (P11) ► | ►**Confusion and misunderstanding:** participants perceive that many consumers do not fully understand what alternative meat products actually are, leading to confusion and difficulties in forming a clear opinion.  ► **Negative attitude** due to lack of understanding and difficulties in forming a clear opinion. |
|  |  | Safety concerns | Concerns over the safety of consuming insect- or cultured meat. | “From a gastronomic point of view, Romanians are traditionalists. They like traditional food, food from Maramureș, homemade meals, the borscht from Moldova, and so on.” (P2)  “The public’s initial reaction will be one of rejection, because most people are conservative. Anything new [...] will be difficult to assimilate.” (P3)  “I also believe that consumers don’t necessarily understand whether these alternative meat products are truly healthy for them.” (P5)  “They might carry pathogenic agents, for example, heavy metals or allergens, so I currently doubt that there are conclusive long-term studies to prove otherwise.” (P12)  “[…] what is produced in the laboratory would have a short-term negative effect on people's health.” (P16) |  |
| 1.2 Do you think that insect-based products and cultured meat will become **part of the regular diet** of Romanians? If yes, in how much time? | **Expected adoption timeline** | Short-term (1-5 years) | Belief that alternative meat products will be adopted within 1-5 years. |  |  |
|  |  | Medium-term (5-10 years) | Expected adoption in a medium-term frame of 5-10 years. | “I think they could become part of the diet, **but it’s hard for me to estimate the timefram**e. It could be 5–10 years, it could be 20.” (P4)  “All of them (note: all three products), I think yes, but as I said, only to a small extent, considering consumers’ perceptions of them. As for when, **I don’t know**, I’d say probably not sooner than 10 years.” (P6) ► | ►**Estimation difficulties:** people find difficult to offer an expected adoption timeline |
|  |  | Long-term (more than 10 years) | Belief that alternative meat products will be adopted in more than 10 years. | “If the situation remains unchanged — meaning things continue at the same pace as now — the process could take quite a long time.” (P1); “it will take time to happen. I’d estimate around 40 years for alternative meat products to become part of Romanians’ diet.” (P15)  “**I’m convinced they won’t**. I’m convinced that Romanians will never eat insects.” (P2)►  “It will probably become a regular part of the Romanian diet over time, but only for some more progressive groups.” (P3)  “It will probably be part of the future, but by then I’ll likely be long gone. I think it will take about 50 years in Romania.” (P6)  “I think it could become part of the diet, but I believe we still have a long way to go. (…) As for when — I don’t know, maybe 10, 20, 30 years… a long period.” (P7)  “**God forbid we end up with such an alternative**, which is by far the most anti-ecological option possible (…) I hope Romanians will stick to their own products.” (P8)►  “Possible, but not likely. (...) Maybe in 15–20 years.” (P9)  “Not necessarily within the next 10 years will they become part of the regular Romanian diet.” (P10)  “I’d say, I don’t know, maybe in 35 to 45 years. I’m 24 years old, I think it’s worth mentioning, so I’m part of **Generation Z** and I have no issue with this, nor do my peers. But I believe it will still take another **two or three generations** for things to change.” (P11)► | ► **Categorical rejection:** firm and absolute refusal to consider insect-based products as acceptable food options  ►**Generational differences** in acceptance: differences in attitudes toward meat alternative between younger generations (e.g., Generation Z) and older cohorts, suggesting that younger people may be more open, while large-scale change would still require generational turnover. |
| 1.3 What consumer **trends** have you observed regarding the **consumption of conventional meat?** | **Decrease** | Sustainability and health concerns | Observed reduction in conventional meat consumption among consumers due to health and environmental sustainability concerns. | “I get the feeling that we are on a downward trend when it comes to meat consumption among a larger segment of the population. (…) It seems that the healthy diet message is reaching an important segment, and we’re starting to see a trend where people consume less conventional meat.” (P10)  “A decrease in meat consumption (…) because it’s healthier, less meat consumption, I’d say, the consumption is lower, but the quality is better.” (P11)  “It’s probably somewhat decreasing, because we’re seeing raw vegans, or vegans, or who knows what else within this globalization paradigm.” (P12)  “If we look 5-10 years ahead, I see more and more people becoming vegetarian.” (P17) |  |
|  | **Maintain** | Culinary and cultural reasons | The influence of longstanding culinary traditions and cultural practices that prioritize the consumption of conventional meat as a staple food. | “More conventional meat is being consumed, and there are orders, if you look online, people are ordering traditional products for Christmas.” (P2)  “Meat consumers are consistent; they don’t change their habits.” (P3)  “I’ve seen an increase in meat consumption, along with a search for **healthier meat products, produced in a cleaner way**.” (P4)►  “I believe Romanians are very fond of meat, especially since we have an entire culinary culture based on a lot of meat and animal fat.” (P7)  “I think conventional meat consumption remains constant.” (P9)  “Conventional meat continues to be consumed on a daily basis.” (P12) | ►**Increased demand for healthier and cleaner conventional meat**: an emerging consumer preference for meat perceived as healthier, with cleaner or more transparent production methods. |
|  |  | Accessibility and price | The ease of access and affordability of conventional meat, along with consumption habits, maintain its popularity. |  |  |
|  | **Increase** | The increase in incomes and the availability of conventional meat | The influence of rising income levels and increased availability of meat on the growing consumption of conventional meat. As income levels rise, consumers often have more disposable income to spend on foods they may view as status symbols or sources of high nutritional value, such as conventional meat. | “Meat consumption has increased significantly, and as a side note, I’d like to mention an interesting fact. As of last year, Romania became the country with the largest number of Angus cattle in Europe, surpassing even Belgium, which had been considered a leader in this field. There is, in fact, an association dedicated to the Angus breed and beef cattle.  Historically, beef has not held a central place in Romanian diets, with pork being preferred, followed by chicken. However, in recent years, beef consumption has increased considerably.” (P1)  “I believe it has increased because, after all, the standard of living has improved in recent years. People have greater purchasing power, and so they tend to buy more meat.” (P6) |  |
|  |  | Promotion of the consumption of animal protein | Social and marketing trends encourage the consumption of animal-based proteins. These trends are often driven by media, advertising, and cultural narratives that associate animal protein with health, strength, vitality, cultural heritage, and traditions. | “And from a qualitative perspective, people are beginning to choose meat from animals smaller than humans, which have a lower growth factor IGF-1, and therefore a lower carcinogenic risk, because the insulin-like growth factor IGF-1 is the main factor being questioned in the consumption of conventional meat. It’s true that in animals larger than humans, this factor is much higher, over 500 units. So, we’re seeing a trend in some segments of the Romanian public, which is beginning to prefer meat from smaller animals, particularly sheep and goats, which have been traditional animals of these people for at least 2,000 years.” (P8) |  |
| 1.4. What consumer **trends** have you observed regarding the **consumption of alternative meat products**? | **Growing interest** | Curiosity and openness toward alternative meat products | Openness toward alternative products out of curiosity. | “I notice that this topic is developing and starting to become more visible.” (P3)  “I think the trend is growing when it comes to plant-based proteins. First, people want to eat healthy.” (P6)  “I think the attitude is one of reluctance, less so in the case of plant-based meat alternative products.” (P5)  “And I’d say there’s interest and curiosity in this area, but for now the options are quite limited and expensive, so the interest mostly remains just that, interest.” (P11)  “I’ve noticed that more and more people are turning to meat alternatives, such as soy or chickpeas (…) for a more diverse diet.” (P12) |  |
|  |  | Ecological and ethical motivations | Motives related to environmental sustainability and ethics. |  |  |
|  | **Occasional consumption** | Occasional or experimental consumption | Occasional or experimental consumption of alternative meat products. | “There is interest and curiosity in this area, but at the moment the options are quite limited and rather expensive, so for now the interest remains just that, interest.” (P11)  “It is possible that there will be a challenge and that there will be high costs at first, at least until people adapt to buying and consuming such products.” (P7)  “Demand will be very low, the prices would be unjustifiably high for a good period of time, which would further hinder the public's familiarity with such products.” (P12) |  |
|  |  | High prices, limited accessibility | Financial or accessibility obstacles to alternative meat products. |  |  |
|  | **Very low consumption** | Cultural resistance | Strong preference for conventional meat. | **“**In Romania, **the consumption of alternative products is a joke, no one consumes them.** There are very few people who want to try them, but not in the sense that they would consume them regularly.” (P2) ►  “I think Romanians are very fond of meat, especially since we have an entire culinary culture based on a lot of meat and animal fat.” (P7) | ►**Perceived insignificance of alternative products**: the perception that alternative meat products are irrelevant or negligible in the Romanian food market, being tested only occasionally by a very small number of consumers. |
| 1.5 What **challenges or difficulties** do **alternative meat products** generate for culinary heritage, rural communities, and conventional meat producers? | **Socioeconomic impacts on rural areas** | - Erosion of culinary traditions | The possibility that culinary traditions may be affected. | “When it comes to culinary heritage, our gastronomy is heavily meat-based. Some traditional gastronomic elements cannot be easily replaced by others. I mean, we can make vegan stuffed cabbage rolls without realizing they’re made from synthetic meat. But we couldn't call those traditional. If we were to certify them as products with known geographical specificity, they wouldn’t fit into those categories.” (P10) | ▼** **No erosion of rural cultural patrimony:** the belief that traditional cultural and culinary heritage will remain stable and largely unaffected by the rise of alternative meat products, as these products serve a separate niche market.  ▼ “In principle, I don’t believe this aspect (i.e., the transition toward alternative meat products) fundamentally affects cultural heritage. There is a well-defined niche for this type of consumption. Even we (…) organize fairs for traditional products with certified names and other similar initiatives. Usually, this niche of traditional products is aimed at a specific audience that consumes such products, and I don't think it will be significantly affected in the near future, regardless of the factors, including artistic or traditional products from rural communities.” (P1) |
|  |  | - Tensions between modern and traditional | Conflicts between modern trends and culinary traditions. | “The people will not give this up. I believe politicians should side with the people and defend national interests. Romania, including through this culinary treasure, I believe we can enrich the global space.” (P2)  “I’m saying that the Romanian household pig bothers them very much. At the national level, we have evaluated the situation of pigs raised in rural household backyards at 500 million euros annually.” (P2)  “Producers and the entire commercial chain, including the promotion of conventional products, will suffer because they will have to adapt and stay alert.” (P3)  “It may also create conflicts at the level of producing companies; it could affect certain communities involved in animal farming.” (P5)  “Our gastronomy is heavily meat-based. Some traditional gastronomic elements simply cannot be easily replaced by others.” (P10)  “It is also clear to us that conventional agriculture is no longer what it was 10 years ago, neither here nor in Europe. There are constant attempts to find alternative methods or products for development. In this context, I don't see a major problem for rural communities. Similarly, in the case of conventional meat producers, the situation largely depends on market demand, but also on input suppliers and the overall food chain. Nevertheless, I don’t believe these producers will face major difficulties, as they are essentially entrepreneurs and can adapt.” (P1)  “Conventional meat producers will suffer because they will no longer have the same level of sales or maintain the same growth trend as before.” (P3)  “Regarding rural communities, I think that in general, especially for many people who have chickens, sheep, and pigs, it would be harder to adapt to this kind of change, and a challenge would be to readapt to another lifestyle and give up these traditions they are used to.” (P7) |  |
|  |  | Reduction in demand for conventional meat | A decrease in demand for conventional meat will affect rural communities. | “It is also clear to us that conventional agriculture is no longer what it was 10 years ago, neither here nor in Europe. There is a constant effort to find alternative methods or products for development. In this context, I don’t see a major problem for rural communities. Similarly, in the case of conventional meat producers, the situation largely depends on market demand, as well as input suppliers and the broader food chain. Nevertheless, I don’t believe these producers will face major difficulties, as they are essentially entrepreneurs and have the ability to adapt.” (P1)  “Conventional meat producers will suffer because they will no longer have the same level of sales or maintain the same growth trend as before.” (P3)  “Regarding rural communities, I think that in general, especially for many people who have chickens, sheep, pigs, it would be harder to adapt to this kind of change, and a challenge would be to readapt to another lifestyle and give up these traditions they are used to.” (P7)  “Clearly, if the consumption of alternative products increases, logically, the demand for conventional products will decrease. Conventional meat producers will suffer because they will no longer have the same sales or maintain the same level of income.” (P3)  “Probably over time, once it truly becomes a trend, the quantity of conventional meat on the market will decrease. And that might become a challenge to address.” (P4)  “It’s clear that conventional meat producers are being affected and have every interest in maintaining their production.” (P9) |  |
|  |  | Economic difficulties for small conventional meat producers | Economic problems for small conventional meat producers. |  |  |
|  |  | Reduction of market and income | Decrease in demand for conventional meat and its impact on the income of conventional meat producers. |  |  |
|  |  | Need for adaptation and innovation | The need for producers to adapt to new market demands. | “Conventional producers must adapt; they will have to improve in the area of marketing.” (P3)  “Small producers, if support programs were introduced, could adopt technologies for processing alternative products.” (P3)  “I think it will be a challenge for conventional meat producers, as they will need to diversify their products and make them more appealing to stay on the market.” (P6) |  |
| 1.6 And what about **opportunities?** | **Economic benefits for producers** | New markets and opportunities for farmers and producers | Economic opportunities for small producers who adopt alternative meat products. | “Value will come out of this competition. Distribution networks will emerge across the entire chain, from producer to consumer.” (P3)  “There are opportunities there, we just need to wait for legislative changes that would allow investors and rural communities to consider this direction.” (P10)  “These communities will likely see the situation as an opportunity.” (P1)  “If they are promoted here, they will grow, in my opinion, they have opportunities here if they diversify their products and services.” (P6)  “(…) we could learn to adapt and discover new industries and new products that could be created.” (P7)  “New tastes, new trends.” (P16)  “ | ▼**Contribution to food security:** role of meat alternatives in enhancing the stability and reliability of the food supply across different geographic scales  “The main benefit would be strengthening food security and resilience at the regional, national, and even continental level.” (P18) |
|  | **Advantages for the environment and public health** | Reducing the ecological footprint | The ecological benefits of meat alternatives through reduced emissions and resource consumption. | “Yes, it could be said that they are healthier, especially the plant-based ones, because they don’t contain cholesterol, they don’t have excessive saturated fats, and they can help achieve a more balanced diet.” (P4)  “There are opportunities to overcome these health and environmental challenges.” (P5)  “There must be no chance of contracting other diseases or compromising the body’s functioning in any way.” (P5)  “I think there are clear advantages when it comes to reducing emissions. Also, reducing agricultural land used for animal feed, so in terms of resources, there are clearly benefits.” (P5)  “Opportunities, yes, because alternative products could potentially be healthier.” (P9)  “Definitely for consumers, because often, if you have a more diverse diet (…) excessive meat consumption is not necessarily good for your health.” (P7)  “I see no advantage in these products. Neither in terms of health nor ecology, because they are highly processed and it’s unclear what they actually contain.” (P8) |  |
|  |  | - Healthier meat alternatives for consumers | Alternative meat products can offer healthier food options. |  |  |
|  |  | - There are no advantages | They are not perceived as having advantages. |  |  |
| **P: Policy and regulation**  (Amato et al., 2023; Bianchi et al., 2018; Fasolin et al., 2019; Foley et al., 2011; Fraser, 2008; Graça et al., 2019; Katz-Rosene et al., 2023; Kwasny et al., 2022; Mancini & Antonioli, 2022; Moritz et al., 2024; Ritchie et al., 2022; Takeda et al., 2023; Tziva et al., 2020) | | | | | |
| RQ2a. **How can fiscal and regulatory policies encourage the adoption of meat alternatives without disadvantaging conventional meat producers?** | | | | | |
| 1. 2.1. Should politicians adopt **strategies to reduce cultural resistance** to the adoption of cultured meat, insect-based products, and plant-based products? If so, what might these strategies be? | **Strategies to reduce cultural resistance** | - Information and education campaigns | Campaigns to inform and educate the public about the benefits of alternative meat products. | “I believe the benefits of these products should be explained very clearly.” (P1)  “(…) Over time, I’ve noticed that the topic has been frequently debated in public space. This issue is often used effectively by both sides of a discussion. Currently, I think the level of public awareness is not sufficient to allow for well-informed decisions. Usually, people reject what they don’t understand, and at the moment, the general attitude seems to be negative.” (P1)  “Politicians need to somehow make future consumers feel more comfortable with these products.” (P3)  “I believe strategies can be proposed to support these alternatives, as long as they fit within a framework that promotes health. (...) Campaigns can be done, and I believe that would be the way to change public perception.” (P4)  “I would promote more the idea of informing citizens, that they should be educated on the advantages and disadvantages of conventional meat and the pros and cons of the alternatives.” (P5)  “We should have a communication plan, a marketing plan for these products that sets the rules of the game.” (P10)  “The difficulty is probably in understanding more precisely what these alternatives consist of and what processes underpin their production.” (P11)  “I don’t think there is a study that proves these products have a favorable nutritional profile.” (P12) | ▼**No need for strategies to reduce cultural resistance:** the view that cultural resistance to alternative meat products should not be actively addressed or diminished through policies or interventions, because these preferences are seen as legitimate and worth preserving rather than changing.  ▼ “Maybe there shouldn’t actually be a policy in this regard right now. I think there should first be a somewhat natural cultural shift.” (P11)  “No, they should not adopt strategies to reduce cultural resistance toward these products.)  “First of all, I don’t think there is a study proving that such products have a favorable nutritional profile. (…) The public lacks the know-how on this issue. There’s also a lack of regulation. (…)  And another very important factor is the sustainability of such an industry on a large scale. We don’t know the processing costs (…). Demand will be very low, and prices would be unjustifiably high for quite a long time, which would make it even harder for the general public to become familiar with such products.” (P12)  “Romania is a national, sovereign, unitary, and independent state. We’ve lived on these lands for over 7,000 years and we have our own culinary traditions. […] I’d still go back to stuffed cabbage rolls, to turkey borscht and pork fat with onions and bread. So I don’t believe politicians should adopt strategies to change the dietary and culinary habits of Romanians. On the contrary, they should protect the existing ones, develop them, and promote them internationally.” (P2) |
|  |  | - Programs to increase public acceptance | Programs aimed at increasing public openness toward alternatives meat products. |  |  |
|  | **Public policy measures for transition** | - Financial subsidies and incentives for producers of alternative meat products | Financial support for alternative meat producers. | “There are food safety regulations that Romania, as an EU member state, must comply with. Otherwise, we risk entering infringement procedures. However, there is also a gray area where minimum and maximum limits are set, and the member state decides what is acceptable. When it comes to hygiene, general norms, and food safety, it is the European Union that sets the rules.” (P1) |  |
|  |  | - Regulations to ensure quality and safety | Legislation regulating the quality and safety of alternative meat products. |  |  |
|  | **Creating a favorable legal framework** | - Legislation to promote sustainability | Regulations to support sustainable practices | “Politicians would not have the moral right to come and override the cultural resistance toward the adoption of synthetic meat.” (P8)  “I’ll give you a concrete proposal: the Romanian state should cover the certification of at least 5 million hectares for organic farming in the mountain areas of Romania, in order to preserve biodiversity in these ecosystems and to provide Romanians with ecological, plant-based and animal-based food from the mountain regions. That would be a legislative change in Romania’s food system that could lead to sustainability.” (P8) |  |
| 2.2 What **aspects** should be considered in the transition toward meat alternatives to **create economic and social benefits** for **small conventional meat producers, local farmers, meat industry workers**, and, in general, **for residents of regions economically dependent on conventional meat production or other specific communities** (religious, ethnic)? | **Support for small producers** | - Transition support for conventional meat producers | Measures intended to help conventional meat producers adapt to changes in the food system. It includes financial subsidies that help them remain viable while reducing conventional meat production and professional retraining programs that enable these producers to gain skills for employment in alternative meat products sectors or other sustainable industries. | “When it comes to alternative products, such as synthetic or other types, they are not included in Annex I. Therefore, at the moment, there is no possibility to support them financially through European funds, in the absence of specific regulation that recognizes their eligibility.” (P1)  “In the area of small producers, and even medium or large producers of conventional products, in my opinion, if government programs were introduced to support new technologies, with certain grants to encourage these producers to adopt technologies for processing alternative meat products, and if they were willing to produce them, it's clear they already have their own distribution chains, and it would be easy for them to introduce these products into their supply chains and thus reach consumers.” (P3)  “Traditional farmers can be supported through consultancy and access to new markets.” (P13)  “The only solution I see in their case, if there were to be a mass shift toward the consumption of meat alternatives, would be programs to support them economically. In other words, some transition programs, if we think about it that way (…) we could provide them with transition funds, which they could use to carry out a sort of rebranding of their product” (P15) |  |
|  | **Economic benefits for regions dependent on the conventional meat industry** | - Job creation in new industries | New employment opportunities in the alternative meat products sector. | “But this (i.e., meat alternative sector) might actually succeed in creating new industries, new jobs, and new products.” (P11)  “(…) the main obstacle is consumers’ willingness, their demand to promote and adopt these alternatives. On the other hand, I’d say this perception may also be justified to some extent, considering that research in the field still needs to advance to prove the health benefits of these products.” (P5) |  |
|  |  | - Integration of small farmers into alternative meat products supply chains | Integrating local farmers into the production of alternative meat products. |  |  |
|  | **Access to quality food for consumers** | - Regulations on fair prices for food products | Policies to ensure that prices for alternative meat products remain affordable. | “A good policy here could be to subsidize them. [...] They should be priced similarly to conventional products, maybe even a bit cheaper.” (P11)  “The products should be available at reasonable prices and take into account local eating habits.” (P13)  “I also believe that consumers don’t necessarily understand whether these alternative meat products are truly healthy for them. So I think these are the main reasons they remain reluctant, they’re thinking about the impact of these products on their bodies.” (P5)  “If we don’t have an information campaign focused specifically on these issues, nothing will happen anytime soon. (...) We don’t have proper information campaigns.” (P10) |  |
|  |  | - Food education and consumer information | Educational programs to inform the public about healthy and sustainable food options. |  |  |
|  | **Respecting cultural and culinary identity** | - Adapting alternative meat products to local preferences | Developing products that respect local specificities and traditions as much as possible. | “From my point of view, we should start with hybrid products that contain both conventional and alternative components. This could ease the transition for the mentioned communities by offering them an acceptable intermediate option.” (P1)  “Yes, there is an increase in plant-based consumption, and there’s a trend of returning to older traditions, the ones our parents and ancestors had, namely, eating meat once a week, on Sundays, as a gastronomic ritual rather than a necessity.” (P8) |  |
|  |  | - Protecting culinary traditions | Preserving culinary traditions in the context of the food transition |  |  |
|  | **Respecting religious and ethnic communities** | - Alternative meat products adapted to religious restrictions | Offering alternative meat products that respect religious and cultural requirements. | “It’s a sensitive topic. From my perspective, we should begin with hybrid products that incorporate both conventional and alternative components. This could facilitate the transition for the mentioned communities by offering them an acceptable intermediate option.” (P1) |  |
| *Supporting policies & fiscal & taxation*  2.3 What **types of instruments** (such as research programs, tax incentives or subsidies, other support programs) could effectively **support innovation and the growth** of the alternative meat products industry?  *[Supporting questions: Can you think of strategies that might help farmers who rely on conventional meat production transition toward this new sector? How do you think the conventional meat sector should be supported to adapt to the changes brought about by the growth of alternative meat products industry?]* | **Research and innovation programs** | - Funding for research in alternative meat production | Financial support for research in the field of alternative meat products. | „Research programs should be prioritized before fiscal measures, and subsidies could come later as a secondary action. But initially, everything related to research in this area is essential. As I mentioned, we are talking about hybrid products or other types of products, which should be supported by research above all.” (P1)  “I think research should be funded a bit, but at the academic level, not at the entrepreneurial level.” (P12) |  |
|  |  | - Public-private partnerships for technological development | Collaborations between the public and private sectors are needed to develop advanced technologies in the food industry. |  |  |
|  | **Financial support instruments for food transition** | - Tax reductions, subsidies for *alternative meat producers* | Tax benefits are granted to producers who invest in alternative meat. | “If it is determined that these products are necessary, one of the methods could be subsidizing along the value chain so that they reach the shelf at a lower price. Another option would be tax reductions, such as VAT, just like it was done for other basic products, where VAT was reduced from 19% to 5%. In this way, both tax incentives and subsidies can be implemented, if that is the goal.” (P1)  “Dedicated funding for startups that want to produce such foods.” (P7)  “First of all, if we initially had subsidy packages that would allow **the coexistence of the two types of sectors side by side**.” (P1)► | ► C**oexistence of conventional and alternative sectors**: public policies or subsidies should enable both the conventional meat sector and meat alternative sector to operate side by side, rather than replacing one with the other, to ensure a smoother transition and minimize disruption. |
|  |  | - Tax reduction, subsidies for *conventional meat farmers transitioning* | Subsidies are granted to traditional farmers to help them adopt sustainable methods. | “**I don’t think they should be subsidized**, and I don’t think they should be supported at all. It’s not our job to create alternative products when **we already have the original**. I mean, you’re asking if we should **fund a copy that will never be equal to the original?** **When can I fund the original to make it even better**? I believe we should subsidize exactly what I said earlier, local production, not alternative products. Local production that is healthy, clean, and ultimately ensures, and will continue to ensure, the survival of the Romanian people.” (P2) ► ► | ►**Rejection of subsidies** for alternative meat products: a strong opposition to financially supporting meat alternatives, arguing that resources should instead focus on preserving and strengthening local and conventional food production, which is viewed as healthier and more authentic.  ►**Trust in local products:** trust and preference for local/traditional products over alternatives, as an implicit barrier to new products. |
|  |  | - Conversion grants for *conventional meat farmers* | Initiatives to retrain farmers from the conventional meat industry. | “Fundamental research should be brought down to the household level, to the farm level.” (P8)  “Financial support, subsidies, assistance in accessing investment programs from European funds, and consultancy. Unfortunately, very few have access to these programs.” (P9, referring to farmers who would need support for transitioning to sustainable practices). |  |
| - 1. 2.4 Should the introduction of a **“meat tax”** (similar to that for sugary drinks) be considered to **promote the sustainability** of the sector? | **Meat tax** | - Introduction of a “meat tax” | A tax imposed on conventional meat products to discourage their consumption. | “I believe there should be no meat tax. We already have too many taxes. I think taxation should be reduced; we should have reasonable fiscal policies (…). I would tax synthetic meat instead. The more you want to introduce synthetic meat, the more I would raise the taxes, and I would use those funds to support conventional products (…). This whole ideology is being imposed primarily by altering the meaning of terms. If you eat tomatoes, that’s a conventional product. Tomatoes have always existed.” (P2)  “I don’t think this meat tax is really necessary; it’s just an attempt to influence the conventional meat sector to make it shrink.  It depends a lot on consumer demand; everything starts from that. You couldn’t impose such a tax if consumer demand for conventional meat is still high, because it would create an imbalance. You’d disappoint both the private sector, the conventional meat producers, and the consumers, who are still the majority. So everything should start with consumers: if consumers demand this tax, then sure, but if not, I don’t think it’s suitable to implement it.” (P5)  “I don’t see why this should happen (i.e., the meat tax).” (P12)  “I don’t think there should be taxes on meat.” (P13) |  |
|  |  | - Mechanisms for redistributing tax revenues | Using the revenues generated from the meat tax to support alternative meat products. |  |  |
|  |  | - Uselessness of a meat tax | Arguments against applying a meat tax, considering it ineffective or inappropriate. |  |  |
| 2.5 There is the *lege ferenda* (the Law on Meat and Meat Products, Senate registration number L494/2023, legislative proposal initiated by deputies from PSD), which **mentions the prohibition of the production and marketing of cultured meat in food products intended for human consumption**. **What is your opinion on this initiative?** | **Ban on cultured meat** | - Support for the initiative | Support for legislation that prohibits the production and marketing of cultured meat. | “I think it’s a very good initiative and it should be improved, because clearly there were some oversights, considering that I see the initiators are from PSD.” (P2) (PSD refers to the Social Democrat Party)  “It doesn't usually happen that I agree with individuals with a communist mindset, but here we are... I can actually agree with these PSD folks for once. So obviously, bravo PSD for finally coming to your senses.” (P8)  “So, if we’re talking about cultured meat, I don’t see it as a bad proposal.” (P11)  “I don’t see what the problem is. I mean, it seems stupid to me, first of all, because you’re blocking an entire industry that could develop and meet the needs of a large part of society. In general, for me, anything related to banning doesn’t sound good, in any field.” (P7) | ▼ **Focus on consumers needs and interests:** the idea that regulations and public policies regarding meat alternatives should be guided by actual consumer demand, health concerns, and the expressed preferences of the population, rather than imposed from the top down. ▼ “Therefore, I also associate this law with the same thing, the interests of the population. If there was demand, if the government considered that there was a small demand for these alternatives and that consumers were concerned about their health, they decided that this should be regulated in the direction desired by the consumer.” (P5) |
|  |  | - Opposition to the initiative | Arguments against banning lab-growing meat, based on reasons such as innovation, consumer freedom of choice, or distortion of the single market. |  |  |
| RQ2b. What impact do international and EU food safety regulations have on the development of alternative meat products in Romania, and how do they affect national food sovereignty? | | | | | |
| *Food industry & Food safety*  2.6 How do **current food safety regulations in the European Union and non-EU countries influence** the development in Romania of cultured meat, insect-based products, and plant-based products, as well as their market?  *[Support question: When we talk about food safety, we mean, for example, food hygiene, general rules for food business operators, and ensuring food safety throughout the entire production and distribution chain.* ***In other words, how do aspects outside Romania influence what happens here?]*** | **External influence** | - The need to comply with EU regulations | The obligation of individuals, businesses, and governments within EU member states to follow the legal standards established by the European Union. | “There are food safety regulations that Romania, as a member state, must comply with.” (P1)  “Compliance with certain standards opens access to various funds.” (P1)  “What happens in the European Union is also mandatory for us in terms of regulations, so there is a direct impact.” (P4)  “(...) the stricter these standards are in terms of food safety, and especially consumer health, the higher the quality of these products will be.” (P5)  “The current European Commission (...) pushed this legislation on synthetic meat and insect-based meat, as if we were living in Africa.” (P8)  “Because at first we think its holding us back […] but at the same time, these regulations require us to follow every step, meaning everything must be clean and hygiene standards must be strictly followed.” (P16) |  |
|  |  | - The necessity to follow the regulations imposed by the European Union | Restrictions on production and distribution imposed by EU and regulations. |  |  |
|  |  | - Opportunities arising from complying with international/EU standards | Development possibilities (e.g., accessing funds) thanks to conformity with the EU/international standards. |  |  |
| 2.7 How do **current regulations** (national, EU, and international) and the trends you observe regarding meat alternatives **influence Romania’s “food sovereignty”?** | **Food sovereignty and dependence on imports** | - Limiting dependence on imports through local alternative meat products | Reducing dependence on imports by supporting domestic production of meat alternatives. | “For example, regarding pork, consumption is around 5 million heads annually in Romania, while we actually produce about 2 million locally, with the rest coming from imports from Poland, Hungary, and Denmark. This is partly linked to the African swine fever epidemic that has affected the sector. However, at present, there is no major issue related to this. Regarding alternatives, it is possible that, as the population gradually accepts alternative products, pork consumption will decrease. This decrease may not necessarily harm local production or imports, but rather result in a diversification of consumption, with an increasing share of alternative products.” (P1)  “Interest in alternative products will increase, and imports of these products will grow as well.” (P16)  “As for food sovereignty, it is a more complex subject, as it also includes food safety strategies. For example, the pandemic or, later, the war in Ukraine made us think more seriously about securing products on the market in case of extreme situations. In this context, food sovereignty assumes a deeper significance. Still, it's hard for me to answer, as we are part of a common market. I don’t know exactly how many meat producers deliver exclusively to the Romanian market and how many export, although this information could be obtained. Looking at both global and national models, I’m not sure how much they influence each other.” (P1)  “Small-scale processing should be encouraged, local production should be encouraged.” (P2)  “I find it hard to believe that we will face food sovereignty problems.” (P3)  “It’s a free market, and food products circulate freely. Production and consumption are more about flexibility than sovereignty.” (P4)  “Romania produces a lot of food, but also imports a lot. In a world with such strong economic interconnections, I don’t know if Romania really has food sovereignty.” (P5)  “I don't know if it affects Romania’s food sovereignty. Right now, I don’t think it does, and I don’t believe it will, if the transition happens naturally in the future.” (P6)  “I don’t see any current regulatory trends that would have a major impact on food sovereignty.” (P10)  “These regulations severely affect Romania’s food sovereignty.” (P8)  “In a world with such strong economic interconnections, I don’t know **if Romania truly has food sovereignty**.” (P5) ►  “I don’t know whether this affects Romania’s food sovereignty. At the moment, I don’t think it does, and I don’t think it will if there is a natural transition in the future.” (P6)  “I don’t see any current regulatory trends that would have a strong impact on food sovereignty.” (P10)  “These regulations severely affect Romania’s food sovereignty.” (P8) | ► **Questioning national food sovereignty**: expressing doubts about Romania’s ability to maintain true food sovereignty in a globalized and economically interconnected world, given its reliance on imports. |
|  |  | - Increasing dependence on imports for alternative meat products | Increased dependence on imports due to a lack of domestic production of meat alternatives. |  |  |
|  |  | - Current regulations do not interfere with national food sovereignty | The existing regulatory framework does not restrict or undermine Romania’s ability to control, manage, and protect its food system. |  |  |
|  | **External regulations and internal control** | - International/EU pressures on national production regulations | External influence on national regulations concerning food products. |  |  |
|  | **Protecting national producers** | - Impact of regulations on small conventional meat producers | Effects of regulations on small-scale conventional meat farmers. |  |  |
|  |  | Protect local producers | Measures to protect local conventional meat producers. |  |  |
|  |  | - Imports/dependence of conventional meat producers | Protecting conventional meat farmers against imports. |  |  |
|  |  | - Imports/dependence of producers of meat alternatives | Protecting alternative meat producers against imports. |  |  |
|  |  |  |  |  |  |
| **E: Environmental impact and animal welfare**  (Chen & Zhang, 2022; de Boer & Aiking, 2022; Foley et al., 2011; Fraser, 2008; Hopkins, 2015; Mroz & Painter, 2023) | | | | | |
| **RQ3. What measures can be taken to develop a sustainable food system in Romania that balances environmental protection and animal welfare?** | | | | | |
| **3.1** Do you think some changes or improvements could benefit Romania’s food system to become sustainable, respecting environmental protection and animal welfare?  *[For example, these changes may target legislation, policies, strategies, action plans, or attitudes.]* | **Legislation and policies for sustainability** | - Introduction of new regulations for environmental protection | New regulations are dedicated to reducing the environmental impact of food production. | “We are already doing this through the program and are required by the regulations. Within the framework of the Strategic Plan for the Rural Development Program, it is stipulated that 40% of the funds allocated to agriculture and rural development should focus on environmental aspects. We have measures for animal welfare in both the plant and animal sectors. We have sectors for plants and animals, as well as eco-schemes, so we are essentially following all the relevant frameworks, including the Farm to Fork Strategy and the 2030 Environmental Strategy, among others.  These measures are working, and we provide complementary payments to offset farmers' income losses because they are no longer producing at their maximum capacity and are reducing production through various measures that encourage environmental protection and respect for animal breeders’ rights.  Additionally, we promote labor equity and require all those accessing our projects to uphold equity principles for their employees, not just animal breeders” (P1)  “Even if they are raised to be slaughtered and consumed later, they must live until that moment in decent conditions, in hygienic and ethical conditions.” (P7)  “At the legislative level, changes should be made so that companies selling certain products in Romania are required to comply with stricter standards regarding the composition of their products. I saw a statistic at the European Union level showing that Romanians often tend to receive chocolate products or even meat that are less healthy in terms of composition compared to those sold in other EU countries. Therefore, stricter regulation of the composition of products entering and being sold in Romania would also ensure greater food safety.” (P15)  “**These technologies**, if we are talking about the production technology for cultured meat and insects, are **extremely non-ecological** by definition (...) As a technology that seriously harms biodiversity and severely impacts the environment through pollution, we are talking about a major environmental concern. First of all, the notion of positive impact should not be associated with these technologies. They are two parallel worlds; **ecology has never been compatible with chemistry**.” (P8)► | ►**Perceived environmental incompatibility of meat alternatives technologies:** the belief that technologies for producing cultured meat or insect-based products are fundamentally incompatible with ecological principles, due to their negative impact on biodiversity and pollution, and therefore should not be framed as environmentally positive. |
|  |  | - Policies and legislation specific to animal welfare | Policies and legislation aimed at ensuring better living conditions for animals. |  |  |
|  |  | - Legislative initiatives to support meat alternatives | Initiatives to promote sustainability in alternative meat production. |  |  |
|  | **Financial support and resources for alternative meat producers** | - Increasing access to resources for alternative meat producers | Funds and other resources are offered to producers of alternative meat. |  |  |
|  |  | - Support for farmers transitioning to sustainable practices | Financial and logistical assistance for farmers adopting more environmentally friendly practices. |  |  |
| **C: Commercial trends**  (Bianchi et al., 2018; Brooker et al., 2022; DeMuth et al., 2023; Petersen et al., 2021; Takeda et al., 2023; Van Loo et al., 2020)  **RQ4a. How can labeling practices for alternative meat products in Romania be improved to ensure transparency, prevent consumer confusion, and support informed food choices?** | | | | | |
| ***Marketing: regulation of advertising and promotion of meat alternatives***  4.1 What do you consider to be the **biggest challenges in regulating the market for alternative meat products**: cultured meat, insect-based products, and plant-based products?  *[You can refer to obstacles from the food industry, consumers, small producers, etc.]* | **Legal challenges** | - Lack of a clear legislative framework for alternative meat products | Absence of specific legislation to regulate alternative meat products. | “In regard to alternative products, such as synthetic ones or others, they are not included in Annex I. Therefore, at this time, there is no possibility to support them financially through European funds.” (P1)  “At the moment, there is no clear legislative framework at the national level in Romania for these products. We don’t know who should authorize them, in what form, or under which authority. These aspects need to be clarified.” (P10)  “It’s hard to say whether a unified standard can be established, because we are talking about very different products: synthetic meat, insects, plant-based products. Each has different regulations and there is no common standard yet.” (P12) |  |
|  |  | - Compliance with EU and non-EU regulations and standards | The need to comply with EU and non-EU regulations and standards. |  |  |
|  |  | - Difficulties in standardizing rules | Challenges in establishing common standards for cultured meat, insect-based, and plant-based products. |  |  |
|  | **Obstacles from the food industry** | - Resistance | Opposition from conventional meat producers toward introducing meat alternatives. | “Clearly, with the emergence of these alternative products, conventional producers and the entire supply and promotion chain will suffer because they will have to adapt and stay competitive.” (P3)  “At the moment, they haven't entered into direct conflict, but in the future, if these alternative products grow, tensions will surely appear between producers.” (P6)  “Probably there will be groups of people lobbying for these alternative products, which will upset the more conservative ones who don’t want to accept them. Divergences will likely arise, at least for a while, until people accept them.” (P3)  “Because the production costs are higher, this automatically increases the final cost for the consumer.” (P4)  “Conventional meat producers are clearly interested in avoiding competition from these alternative products.” (P9)  “I don’t know what the production costs of alternative meat are, maybe in the long run they’ll be more cost-effective, but right now the costs are clearly high, especially due to the technology.” (P10) |  |
|  |  | - Conflicts | Tensions between conventional and alternative meat producers due to divergent interests. |  |  |
|  |  | - Costs | Significant expenses are required to implement changes for alternative meat production. |  |  |
|  | **Consumer reactions** | - Lack of trust | Consumer reluctance toward alternative meat products due to uncertainties. | “People usually reject what they do not know.” (P1);  “There will certainly be obstacles because this is part of human nature. When change occurs, there is a certain resistance to it, as we are used to living in a certain way, and change can be difficult. This applies not only to food but to almost every aspect of our lives. However, I believe that **effective and accurate information** is key to overcoming these challenges. I could make a comparison with electric cars. If we look back ten years ago, electric cars were seen as an abstract concept, unsustainable. But now, the majority of Romanians would like to own an electric car, and many have already bought one, and they are now seen on the streets. It’s viewed as something normal, you know? The information was correct, the advantages were presented, but also their disadvantages. And this applies to alternative meat products as well. That’s why I believe that the information provided to decision-makers should be as accurate as possible.” (P5)►  “Consumer reluctance seems to me to be the main challenge. In my opinion, it would be absurd for the private sector and decision-makers to try to promote alternatives if there is no demand from the population. That is, the population must first express a demand, and only then will private actors try to meet these needs, because ultimately, they operate on the idea that the customer is always right. And the government must respond to these demands; it would be wrong to make decisions that go against the interests of the people. So, the main obstacle is the consumers’ willingness and demand to promote and adopt these alternatives.” (P5)  “I believe the biggest challenge is to overcome the mindset of the majority." (P6)  “I think the biggest challenge is actually the resistance people have to such things and how far removed it seems from our culture to consume insects.” (P7)  “There is a component of traditionalism in meat consumption among the Romanian population.” (P10) | ►**Importance of effective and accurate information for behavior change**: the belief that providing high-quality, reliable, and transparent information to the public is essential to reduce resistance to change and to facilitate acceptance of innovations, including alternative meat products. |
|  |  | - Concerns about food safety and health | Worries about potential health risks associated with alternative meat products. |  |  |
|  |  | - Cultural resistance and psychological barriers | Cultural opposition and mindsets that discourage the adoption of new products. |  |  |
|  | **Challenges for small producers** | - Difficulties adapting to new technologies | Challenges for small producers in adopting new technologies and standards. | “Probably the difficulty lies in understanding more precisely what the alternatives consist of and what are the processes behind the production of these alternatives. Given that the Romanian producer, the typical Romanian producer, focuses on small-scale production and sales, they don’t really think much about other alternatives.” (P11)  “I believe the difficulty might be the increasing competition in a market that I don’t necessarily see as similar, because people will seek both alternatives and natural, functional sources of meat. Probably, over time, once it becomes an actual trend, the amount of conventional meat on the market will decrease. And that could become a challenge to address.” (P4) |  |
|  |  | - Lack of resources to compete with large producers | Small producers often face limited financial resources compared to their larger competitors. |  |  |
|  |  | - Need for financial support and market access | Small producers need financial support and market access for meat alternatives. |  |  |
|  | **Costs and technological infrastructure** | - Investments needed for technology development | Large sums required to develop alternative production technologies. | “There are high costs, I don’t know what the production costs of their alternative meat are, maybe in the long term they are more profitable than conventional meat products.” (P10) |  |
|  |  | - Logistical and distribution challenges | Difficulties in managing the transport and distribution of alternative meat products. |  |  |
|  | **Ethical and sustainability aspects** | - Ethical controversies regarding cultured meat and insect-based products | Ethical discussions about using living organisms and synthetic processes. | “If we’re talking about the technology for producing synthetic meat and insects, they are extremely non-ecological by definition (…) Being a technology that severely affects biodiversity, being a technology that seriously harms the environment in terms of pollution.” (P8)  “We know very well that we have this extremist, nationalist wave, which has a very strong stance against these products.” (P10) |  |
|  |  | - Controversies about actual sustainability of alternative maet products | Debates on the true environmental and sustainability impact of these products. |  |  |
| **RQ4b. What role does media coverage, across television, social media, blogs, and political discourse, play in shaping Romanian consumers’ perceptions, attitudes, and trust toward alternative meat products?** | | | | | |
| **4.2** What is your opinion on the **media coverage** (TV shows, blogs, political speeches, social media, etc.) of cultured meat, insect-based, and plant-based products? Please consider whether this **coverage ensures correct consumer information.** | **Media framing** | - Accuracy | The accuracy of information provided about the benefits and risks of alternative meat products. | “No, it does not ensure. From what I have heard, as an individual citizen, I have only heard negative reactions. So far, I haven't heard a single positive one.” (P1)  “I don’t know if the information is always correct. I’d say that many times it is distorted.” (P6)  “There are always risks when new biological or technological developments appear, just like during the pandemic with the very rapid emergence of those vaccines.” (P9)  “It depends on the source of media coverage. We know very well that both politicians and mass media, social media sources often spread misinformation on many topics.” (P10)  “In Romania, the media coverage is flawed and rather contributes to misinformation.” (P11)  “The media coverage, the way it has been done so far, didn’t seem particularly fair to me.” (P4) |  |
|  |  | - Bias | The level of detail and truthfulness of information available on different platforms. |  |  |
|  |  |  | The need to provide correct and balanced information about alternative meat products. |  |  |
|  | **Objectivity *vs.* commercial messaging** | - Influence of commercial interests on media messages | Commercial interests may distort media messages to promote alternative meat products. | “It doesn’t ensure proper information. The purpose of the media coverage is precisely this marketing we’re talking about, this fake marketing meant to change human behavior, and I believe this kind of media coverage should somehow be regulated, so that when there are deviations, they can be sanctioned. The citizen has the right to be properly informed and to make decisions knowingly.” (P2) |  |
|  |  | - Exaggeration | Promotion that presents the benefits of products in an overly optimistic or unrealistic way. | “I think it’s more about what the consumer wants to hear, people might be more drawn to the idea of spectacle when watching TV than to actual information.” (P5) |  |
|  | **Impact on consumer perceptions** | - Shaping public perceptions | Media bias either in favor of or against alternative meat products. | “I’d say that people are not well informed, and perhaps their attitudes and public perception are even intentionally influenced.” (P6)  “I’ve hardly seen any media coverage of these products, the only mentions were negative and came from extremist parties.” (P9) |  |
|  |  | - Possibility of misleading consumers | Influencing how the public perceives alternative meat products. |  |  |
|  |  | - Creating a positive or negative image | Information that might confuse the public and influence perceptions of alternative meat products. | “At the moment, the negative publicity around these products is much stronger, and we don’t have proper information campaigns. So, I don’t see how they could make their way into the regular Romanian diet.” (P10)  “Because of media coverage of issues related to animal welfare and sustainability, people have become more aware of animal farming practices on small plots of land with high density, their transport, and especially their slaughter. For example, the local slaughter points that used to operate during Easter fairs, where lambs were traditionally slaughtered, have mostly disappeared, precisely because many people began to feel a sense of repulsion toward this activity.” (P18) |  |
|  | **Diversity of media platforms** | - The role of social media, TV shows, and documentaries | Mass media can influence how the public sees alternative meat products (as beneficial or not). | “From what I’ve observed in the media and in public reactions, they are quite negative. There is a strong reluctance among ordinary people to consume alternative sources of meat, let’s say, those that are chemically processed and so on. A few years ago, there was even a heated debate regarding a proposal at the European Union level to introduce a certain type of insect-based flour.” (P14) |  |
|  |  | - Shaping public opinion through different media channels | Platforms influence perceptions by promoting alternative meat products. | “If situations where animals are not kept in proper conditions are sufficiently publicized, people are horrified.” (P10) |  |
|  |  | - Influence of blogs and political speeches | Blogs and politicians play a role in informing and influencing public opinion on alternative meat products. | “(…) politicians tend to use this subject more as a stepping stone or a means of promotion, as it is a sensitive issue. But they do not approach it in a positive way. Additionally, much of the information available on blogs and other sources is merely personal opinions. I, for example, as a consumer, would like to have access to studies and clear scientific information about these products. Why should we use such products? Until now, I haven't heard an argument based on such information” P1  “I believe that, in general, people focus more on sustainability, as there are animal welfare measures, including in our country, although they are voluntary schemes, not mandatory. Many farmers adopt them, but I think sustainability in agriculture, in relation to environmental impact and similar factors, is much more important than animal welfare.” P1  “Blogs, TV shows, social media, and so on, all of these are simply trying to **provide consumers with content, not to inform them**. So I wouldn’t say that all of these have a positive role when it comes to informing consumers.” (P5) P5 **►** | **►No role in informing:** the perception that popular media (blogs, social media, TV shows) prioritize attractive or sensational content over accurate and factual information, thereby failing to properly inform consumers. |
| ***About labelling***  **4.3** How could the existing **regulatory framework be improved, or what new legal provisions should be adopted regarding the labeling** of cultured meat, insect-based products, and plant-based products, to ensure that consumers receive clear, transparent, and non-misleading information when choosing alternative meat products?  *[Support: What are the most important aspects you or your party take into account when naming new food products? When you give them a name?]* | **Labeling transparency** | - Ingredients and nutritional values | Clear detailed breakdown of product composition and nutritional values to properly inform consumers about alternative meat products. | “ (…) there should be very clear and detailed information provided to the public, both about the advantages and the risks. If at some point there is a need to label these products, it must be done in a clear and distinct way, just like it’s done with organic products or those with controlled origin, and so on. This is important both to ensure proper and complete consumer information and to prevent fraud.” (P1)  “The ingredients, of course, the complete and exact list, and also a logo or a distinctive sign. There’s nothing wrong with that; it’s already a common practice for any traditional European product, and so on.” (P1)  “It should clearly state that they do not contain animal meat, but otherwise **I don’t know**.” P4 **►**  “The label, first of all, must inform about the product — whether it’s synthetic meat, insect-based, or plant-based. So that the buyer can choose what they want to purchase.” (P6)  “First of all, the title ‘meat alternative product,’ and secondly, what the main ingredient is — the one that makes up the largest proportion of the product.” (P7)  “It would be much more beneficial from a marketing perspective if we didn’t associate these products with existing ones on the market.” (P10)  “I think it should be mentioned that there is a difference compared to the original food the alternative is based on.” (P11)  “I believe the clear composition, the source, the processing used, and allergenic ingredients should be mentioned.” (P13) | **►Lack of knowledge about labeling requirements**: expressing a lack of clarity or confidence about what additional information should appear on alternative meat product labels beyond basic animal-free content, reflecting consumer or stakeholder uncertainty. |
|  |  | - Product type | Clarifying whether the product is cultured meat, insect-based, or plant-based to avoid consumer confusion. |  |  |
|  |  | - Raw material sources | Specifying the origin of ingredients (local, EU, non-EU) to support transparency. |  |  |
|  |  | - Production method | Explanations of the production methods used, such as cultured meat, to enhance transparency. |  |  |
|  |  | - Ecological impact | Data on the product’s effects on the environment to help consumers make informed choices. |  |  |
|  |  | - Health/ safety | Clear information about the benefits and potential health risks associated with consuming the product (e.g., allergens). |  |  |
|  | **Regulations to prevent misleading practices** | - Ban on the use of traditional terms | Prohibition on using terms like “meat” to avoid confusion with conventional products. | “You don’t have the moral or agronomic right to call cultured meat ‘meat.’” (P8)  “First of all, you don’t have the right to use the expression ‘meat alternative product’ (…) it creates a predisposition in me.” (P8) |  |
|  | **Quality certifications** | - Implementation of recognized certifications | Applying well-known European and national certifications for the quality of alternative meat products. | “If insect-based and cultured meat products are not developed to have a similar appearance and taste to conventional meat, and at the same time don’t obtain all the production labels that show consumers they are healthy, I don’t think they will go very far.” (P5) |  |
|  | **Consumer education and information** | Public information campaigns | Initiatives to educate the public to understand new labels and product types better. | “Of course, information campaigns should be carried out, but I **don’t think anyone expects them to have immediate results.”** P9► | ►▼**Lack of trust in public information campaigns**: participants express doubts about the effectiveness or honesty of public information campaigns related to alternative proteins, suspecting they may not deliver unbiased or credible messages, which undermines their potential impact.  ▼ “ (…) we do not have correct information campaigns.” P10 |
| T: Technological development and innovation  (Chiles, 2013; de Amstalden, 2024; Etzkowitz & Leydesdorff, 2000; Tziva et al., 2020) | | | | | |
| **RQ5a. What technological and infrastructural challenges hinder the growth of the alternative meat sector in Romania, and how can they be addressed?** | | | | | |
| 5.1 What are the main **challenges and barriers** in the current **technologies and infrastructure** for the production of alternative meat: cultured meat, insect-based products, and plant-based products? Let’s take them one by one.  a) First, what are the **challenges and barriers?**  b) Now, what are the **opportunities** related to accessing technologies and infrastructure? | **Technological change** | Technological challenges | Technical difficulties in producing alternative meat from various sources. | “(…) probably the technologies for producing alternative meat products require large investments. This period, as we see, is a bit uncertain, and probably in the West, the scientific basis of a crisis is being laid, one that might affect us in the future. God forbid, but it seems a wave of economic downturn is coming from the West in major countries, and it will probably affect our country too. In that case, I don’t know if producers will be willing to try uncertain products. And it’s unclear whether they will bring back economic value, in terms of profit for them. That’s why I don’t know if they’ll have the courage to try adopting these technologies in the short term, so that would be a big challenge. You mentioned that certain bans on these products have already been adopted, that is clearly an insurmountable barrier. There is also the barrier of how the majority of consumers position themselves, they tend to be conservative, very reluctant to try them. So, a social barrier, a legislative barrier, and an economic barrier... That’s already three things that could stop the progress of these products in the short term.” (P3)  “Well, the current technology and infrastructure do not yet provide the greatest benefits when it comes to imitating conventional meat, in terms of texture and taste, and that makes consumers reluctant. Also, as I mentioned, these products tend to be **ultra-processe**d, which is where the debates stem from about how healthy they truly are compared to conventional meat. In fact, this is the biggest challenge that meat alternatives have to overcome through technology: to address the health concerns first, and then those related to appearance and taste, so they can earn consumer trust.” (P5) **►** | **►▼Association of alternatives with ultra-processing:** the perception that meat alternatives are highly processed and therefore less healthy.  **▼** “(Note: alternatives) are highly processed and it’s not clear what they contain.” (P8)  “I see a great deal of reluctance among ordinary people to consume alternative sources of meat, let’s say, those that are chemically processed and so on.” (P14) |
|  |  | Costs of infrastructure/technology | Significant expenses associated with the infrastructure needed for alternative meat production. |  |  |
|  |  | Problems scaling up | Challenges in scaling up production and distributing alternative meat products on a large scale. |  |  |
|  | **Opportunities for innovation** | Development of new technologies that reduce production costs | Innovative technologies that can lower production costs for alternative meat products. | “As for the technologies, **I can’t answer exactly because I don’t know the details of the process or what type of machinery and equipment are needed**. However, I can tell you that, regarding accessibility, Romanian farmers are currently using the latest technologies available. Of course, not all farmers, but at least the large ones and those in the agri-food industry are benefiting from them. The European Union's openness has enabled a wide range of customized technologies for each of them, whether we’re talking about robots, drones, or other equipment that, a few years ago, were seen as cutting-edge technology. These are now within their reach. I would highlight here the openness of farmers and industry entrepreneurs to these innovations.” (P1)**►**  “Possibly the low costs or the substitution of meat for vegans.” (P13)  “So the connection with those who have already adopted these technologies is indeed an opportunity — to help our economic agents import the know-how from there, both the hard and the soft aspects.” (P3) | **►Lack of knowledge about alternative meat production:** respondents express a lack of understanding regarding the specific technologies, processes, or equipment required for producing alternative meat products, highlighting a technical knowledge gap. |
|  |  | Access to funding for the necessary infrastructure | Dedicated funds to develop the infrastructure required for alternative meat production. |  |  |
|  |  | Innovations in the distribution and logistics of alternative meat products | Development of efficient logistical solutions for distributing alternative meat products. |  |  |
| **RQ5b. What role do intellectual property rights and cross-sector collaboration play in fostering or limiting innovation in Romania’s alternative meat industry?** | | | | | |
| 5.2 When we talk about intellectual property, we can think of patents, trade secrets, trademarks, and copyrights that protect innovations and the creation of new products in this field. **Do you think intellectual** property for cultured meat, insect-based products, and plant-based products **plays an essential role in stimulating innovation in the alternative meat products sector?** | **Intellectual property: barrier vs. accelerator** | Intellectual property as a potential barrier for new market entrants | Intellectual property can make it harder for new producers to enter the alternative meat products market. | “Yes, as in any other field. Because once you have intellectual property over a certain product, you can sell the rights or commercialization rights, and so on. It is in your best interest to start developing as many products as possible to maximize profits.” (P1)  “They invest money, and naturally, they should have the opportunity to recover that money. Someone can’t produce a product without incurring costs and distribute it without gaining any advantage from their work. Since these are new products, it is very challenging because, compared to traditional products, it’s difficult to adapt and, at the same time, capture the market. Essentially, you need to innovate and conquer the market simultaneously. That’s why I believe that those who venture into this area to innovate are true pioneers. And, in a way, they should be recognized, including for intellectual property rights, which would allow them to do so.” (P1)  “I don’t think it plays a role in stimulating innovation. I think it’s just another way to extract more money. I patent the product, then ask for money for the patent. As if trying to destroy my food security and the health of the population wasn’t enough, you want to do it with my money? I don’t agree with that either.” (P2)  “Definitely yes, because intellectual property, as far as I know, generates revenue for the research groups working on patents and for putting them at the disposal of economic agents who will then profit from them.” (P3)  “I believe intellectual property rights are a barrier, because no one else can use the product or the technology.” (P13)  “Yes, absolutely, there are consumers who see a certain brand, for example, and say they actually trust it, and that can influence consumption. So yes, in the end, intellectual property does play a role in shaping consumer perception.” (P5) |  |
|  |  | The possibility that patents may limit access to technology | Patents can prevent the widespread distribution of certain technologies to produce alternative meat products. |  |  |
|  |  | The importance of trademarks and copyrights | Trademarks and copyrights are essential for building trust in alternative meat products. |  |  |
| 5.3 Does **collaboration** between public authorities, companies, universities, and research institutions play an important role **in stimulating innovation for the development of alternative meat products?** How could this collaboration be encouraged?  *[What is missing, what could be improved?]* | **The importance of collaboration in stimulating innovation** | The role of public–private partnerships in technological development | Collaboration between government and the private sector supports the development of technologies for alternative meat products. | “As far as I’m concerned, I can tell you exactly what I work on. At the European level, there is a system called AKIS, an acronym derived from the English term "Agricultural Knowledge and Innovation System." Under this umbrella, we aim to bring together the actors you mentioned, public companies, universities, research institutes, and explore together any interesting ideas for agriculture, including those related to alternative meat products. We have no issues in this regard, and we try, through these working groups, to focus on specific topics, to produce and disseminate either production techniques, innovative ideas, best practices, or simply answers to the questions of those interested.” (P1)  “(…) from close collaboration come public policies, market acceptance, and funding. So basically, only collaboration will make it possible for these products to end up on store shelves for people in the future.” (P3)  “This communication framework must be created; this interest must be understood, just as I said earlier, and each party should contribute with their own resources, financial, regulatory, technological investment, and so on. So, this framework is absolutely necessary. Without these actors and without them acting in an organized way to meet the population’s needs, I don’t think major progress will happen.” (P5)  “Collaboration between public institutions, companies, and academia is beneficial in any field.” (P6)  “The university and research institutions need to go directly into households and farms to understand their needs.” (P8) |  |
|  |  | Collaboration between universities and companies for applied research | Partnerships between academia and industry facilitate the application of new technologies for alternative meat products. |  |  |
|  |  | The importance of public institutions in supporting innovation | Public institutions play an important role in supporting research and the development of alternative meat products. |  |  |
| 5.4 We know that an **open-source and non-exclusive license** is one that allows the **invention to be used freely**, without fees, by anyone. How do you think **openly sharing technologies and data through open-source and non-exclusive licenses** could **accelerate innovation** and the growth of the alternative meat products sector?  **a) What benefits** could this bring for companies, researchers, and consumers?  **b)** What about **problems or disadvantages** that can be generated? | **Benefits of open-source licenses for innovation** | Accelerating innovation through free access to technologies | Open-source licenses allow free access to technologies, thereby accelerating innovation in the alternative meat products industry | “This topic is a **bit beyond my expertise**; I’m not very familiar with the IT field, especially with open-source and non-exclusive areas. I understand the question, but I don’t think I can give you a very coherent answer here.” (P1)**►**  “Clearly, a technology that can be adopted freely, without being conditioned by paying certain amounts, will be much more appealing to an operator starting a small business — because they can begin with less money — compared to a technology that requires large investments and comes with a high degree of uncertainty regarding profit. So, in my opinion, an open-source license would be a good idea for promoting these products.” (P3)  “If the data and information are open, development in this direction progresses faster.” (P6)  “Maybe that’s exactly the point, in my view, when there are open-source licenses, you give more people the opportunity (…) to develop those technologies.” (P7)  “Reducing research costs and allowing quick access to solutions, thus stimulating rapid growth.” (P13) | **►Lack of knowledge on open-source concepts: participants** acknowledge limited familiarity with the concepts of open-source or non-exclusive licensing, indicating a gap in knowledge about intellectual property models relevant to alternative meta technologies. |
|  |  | Reducing costs for companies and researchers | Companies and researchers can save resources by using open-source technology. |  |  |
|  |  | Access to technologies for smaller companies and start-ups | Small companies and start-ups benefit from access to technologies that would otherwise be inaccessible. |  |  |
|  | **Disadvantages and risks of open-source licenses** | Potential reduction in private investment in research | There is a risk that private investors will stop funding research if technologies are freely available. | “I see this field as being in a pioneering stage. In the case of innovation towards new products and technologies in this sector, which is still in its early phase, I believe those who innovate and produce viable solutions must be somehow rewarded. Later on, we can adopt models like Open Source or benchmarking, which are essentially methods for improving an already existing process. These are not entirely new processes, and I fully agree with this approach.” (P1)  “**A license** is, by its nature, **an attribute of the industrial sector,** I don’t want to encourage an industrial form of organic agriculture.” (P8)**►**  “**I’m cautious** when it comes to open-source licenses. **I’m not sure** these open-source licenses could actually accelerate innovation.” (P9)**►**  “Open-source licenses tend to homogenize a sector. If everyone has access to the same types of information, then no one has an advantage over their competitors.” (P10) | **► Criticism of industrial licensing in sustainable agriculture**: the perception that applying formal licenses and intellectual property frameworks, typical of industrial sectors, is inappropriate or even harmful when applied to ecological or sustainable farming practices, which traditionally rely on openness and community knowledge-sharing.  **►Skepticism toward open-source innovation:** expressing doubt that open-source licensing models can truly stimulate innovation, reflecting a critical view of their effectiveness or sustainability in the agri-food sector. |
|  |  | Risk of improper use | Technologies may be used incorrectly, which could affect the quality of products on the market. |  |  |
|  |  | Difficulties in maintaining a competitive advantage | Companies may lose their competitive advantage if alternative meat products technology is available to everyone. |  |  |

Note: *A downward arrow (▼) indicates that the inductive code emerged directly from a quote presented exclusively in the sixth column. A horizontal arrow (►) indicates that the inductive code is associated with a statement that also contributed to a deductive code/subcode, as presented in column five.
